# Advances towards Cell‐Specific Gene Transfection: A Small‐Molecule Approach Allows Order‐of‐Magnitude Selectivity

**DOI:** 10.1002/chem.202104618

**Published:** 2022-06-17

**Authors:** Thies Dirksmeyer, Paul Stahl, Cecilia Vallet, Shirley Knauer, Michael Giese, Carsten Schmuck, Christoph Hirschhäuser

**Affiliations:** ^1^ Institute of Organic Chemistry University of Duisburg-Essen 45117 Essen Germany; ^2^ Institute of Biology University of Duisburg-Essen 45117 Essen Germany

**Keywords:** biotin, cancer cell targeting, cell-penetrating peptides, gene technology, small molecules

## Abstract

A transfection vector that can home in on tumors is reported. Whereas previous vectors that allow moderately cell selective gene transfection used larger systems, this small‐molecule approach paved the way for precise structure‐activity relationship optimization. For this, biotin, which mediates cell selectivity, was combined with the potent DNA‐binding motif tetralysine‐guanidinocarbonypyrrol via a hydrophilic linker, thus enabling SAR‐based optimization. The new vector mediated biotin receptor (BR)‐selective transfection of cell lines with different BR expression levels. Computer‐based analyses of microscopy images revealed a preference of one order of magnitude for the BR‐positive cell lines over the BR‐negative controls.

## Graphical abstract

**Picky for cancer cells**: A small‐molecule transfection vector containing a strong DNA binder and a targeting moiety shows low toxicity and a tenfold selectivity for cell lines expressing biotin receptors (BR). The binder contains the tailor‐made guanidinocarbonypyrrol (GCP) motif. A tetrapeptide of only four GCP‐modified lysine residues is usually sufficient to achieve transfection. Addition of an appropriate linker deactivates the vector. Further attachment of a biotin label allowed BR‐rich cancer cell lines to be targeted.

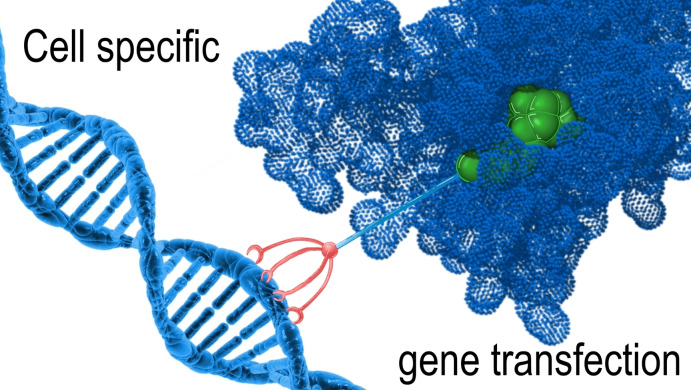

Gene transfection is playing an increasingly important role in the field of biomedicine and can pave the way towards new treatment strategies for various diseases.^[1]^ The swift development of vaccines against SARS‐CoV‐2^[2]^ illustrates the potential of this concept in a dramatic way. Further promising progress occurred in the treatment of cancer^[3]^ with artificial vectors. For this application in particular, a need for potent, biodegradable and cell type‐specific vectors is obvious.^[4]^ However, selective targeting of cancer cells still represents a tremendous challenge.^[5]^ Consequently, the number of vector systems that are able to address specific cancer cell lines selectively is limited. Nevertheless, a variety of different targeting moieties and concepts have been implemented, leading to moderately selective transfection vectors. Among the bioactive homing molecules employed are folic acid,[^6^, ^7^] biotin,^[8]^ avidin^[9]^ or galactose.^[10]^ Furthermore, cells with high levels of reactive oxygen species (ROS)^[11]^ have been targeted. With these systems, transfection efficiencies were achieved, which were up to four times higher for the targeted cancer cells than for suitable controls. In this article, we describe the first small‐molecule transfection vector (**1**) with a selectivity that is an order of magnitude higher for cancer cell lines expressing biotin receptors (A549 and HeLa) than for biotin‐receptor‐deficient controls (CHO, HEK293T, HCT116).

Most of the aforementioned examples from literature consist of multiple components and employ units for DNA binding, which are significantly larger than the associated targeting unit. For example, Wang and co‐workers^[6]^ reported a poly(β‐amino ester)‐based vector, which was functionalized with folate as a targeting moiety. This vector system was able to transfect HeLa cells with a transfection efficiency of up to three times higher than vectors without folate. HeLa cells exhibit high levels of folate‐receptors, which are recognized by the label. Interestingly, the transfection selectivity was reported to be dependent on the folate density on the polymeric scaffold. Increased folic acid loading led to increased hydrophobicity and thus larger polyplexes with decreased transfection efficiency.

In more general terms, such a tradeoff between selectivity and efficiency is to be expected, as increasing the number of targeting moieties per DNA binder is likely to reduce DNA‐binding capacity. A small‐molecule approach in which the cellular recognition unit is combined with a very strong DNA binder of similar size addresses this issue. Due to the strong binding interaction less DNA binder can be used per targeting moiety, thus increasing the relative amount of targeting moiety in a DNA‐vector polyplex. Furthermore, a small‐molecule approach allows for addressing hydrophobicity issues by synthesis.

As shown in Figure 1A, we set out to implement the strong DNA binder tetralysine‐guanidinocarbonylpyrrol (tetralysine‐GCP, red) onto an easily modifiable peptide platform (blue),^[12]^ thus connecting to the selectivity‐mediating targeting moiety biotin (green). Tetralysine‐GCP, though unselective, was reported in 2015 and can serve as the smallest peptidic transfection vector. It consists of only four lysine moieties,^[13]^ modified with the tailor‐made GCP anion‐binding motif.[^14^, ^15^] The interaction between the GCP group and the DNA phosphate backbone is made up of electrostatic interactions between the guanidine cation and additional hydrogen bonds contributed by the pyrrole moiety (Figure 1B).^[15]^ It is likely that the strong anion‐binding characteristics of the GCP moiety together with its comparatively low basicity enable endocytotic uptake through increased interactions with negatively charged groups on the cellular membrane, as well as a proton sponge mediated endosomal escape, respectively.^[14]^ As a binding motif tetralysine GCP is of similar size as biotin (vitamin H, Figure 2), that is, the targeting moiety (green). Internalization of biotin is mediated by different biotin receptors,[^16^, ^17^] which are expressed in higher levels in many cancer cells. Biotin receptors therefore represent a valuable target for bio‐medical applications.^[16]^ By exploiting the excellent anion‐binding properties of the GCP group, **1** can condense DNA into sufficiently packed polyplexes, which are taken up preferably by biotin receptor‐positive cell lines (Figure 1C).

**Figure 1:**
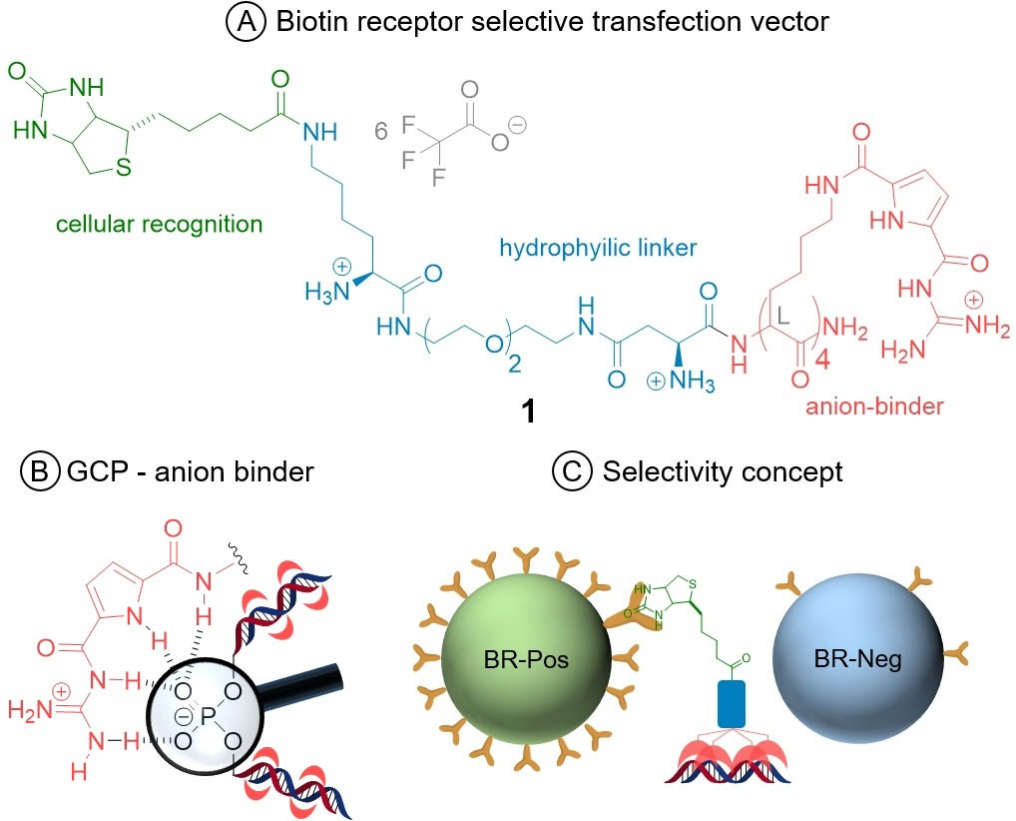
A) Cell‐specific gene‐transfection vector comprising the cellular‐recognition unit biotin (green), a hydrophilic linker unit (blue) and the tetralysine‐GCP DNA binder (red). B) Supramolecular interactions between the GCP cation (red) and the phosphate backbone of DNA. C) Selective DNA delivery by interaction with the biotin‐receptor‐rich cell surface.

**Figure 2:**
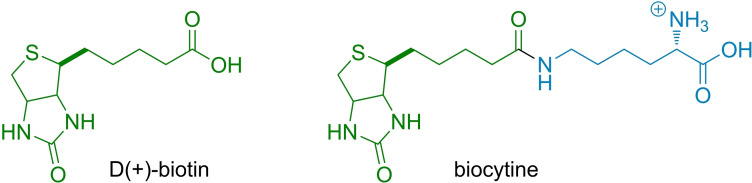
Structural formula of d(+)‐biotin and biocytine.

The development of vector **1** is illustrated in Table 1. In order to assess structure–activity relationships (SAR), we analyzed the minimal concentration needed to achieve transfection in steps of 100 μM, the toxicological profile and the transfection efficiency. To get swift access to toxicity data for SAR analysis, the compounds listed in Table 1 were evaluated in an MTS‐based cell‐viability assay, and transfection efficiencies were analyzed by CellProfiler® on HeLa cells (see the Supporting Information for details).^[18]^ Entry A shows the performance of the tetralysine‐GCP lead structure in this assay. Amide formation on the N terminus reduced the net charge by 1, and acetylation at this position led to an increased concentration needed for successful transfection (Table 1, Entry B). Finally, the descent in transfection efficiency to 5.9 % highlights the relevance of a fifth positive charge.^[19]^ Apparently, this reduction of positive charges impedes DNA condensation and must be compensated for.

**Table 1:** Semi‐quantitative structure activity relationship analysis of different vector precursors tested in HeLa cells.

	Chemical structure	Conc. [μM]^[a]^	Toxicity.^[b]^	Efficiency [%]^[c]^
A^[d]^	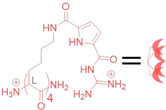	150 (min.) 300 (opt.)	low high	3.2 48
B	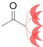	400	low	5.9
C	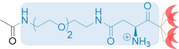	200	low	0.08
D	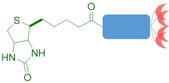	150	low	1.8
E	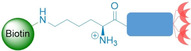	100 (min.) 150 (opt.)	low	6.9
	**1** vector			
F	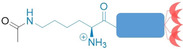	*no transfection*	low	0
	**2** control

[a] Describes the minimal concentration needed to observe successful transfection during microscopy. [b] Qualitative description of cellular toxicity in the concentration range optimal for transfection as evaluated by MTS assay: Low=cell viability ≥70 %, high=cell viability <70 %. For full data see the Supporting Information. [c] Transfection efficiency determined by CellProfiler® readout. [d] Given data was determined as described by Li et al.^[13]^

To account for that, an ethylene glycol‐asparagine linker was introduced. The ethylene glycol moiety counteracts the low water solubility of biotin in aqueous media^[20]^ and the asparagine moiety provides the additional positive charge to the binding unit. The introduction of multiple charged amino acids to increase solubility was avoided as it might interfere with the genetic material.^[21]^ Additionally, the linker unit provides flexibility and some distance between the DNA binding unit and the biocytin label, enabling the latter to protrude from the vector‐DNA polyplex and enhance binding availability. Attachment of the acetyl‐capped linker (entry C, Table 1) led to a vector which transfected at comparably low concentrations (200 μM) and exhibited low toxicity in HeLa cells. However, the transfection efficiency was insignificant (0.08 %) and did not increase at higher concentrations. This general profile, however, was desirable, as good transfection efficiency without the biotin label would antagonize the selectivity of a labeled vector. Hence, the introduction of biotin was the next step in the development. The resulting vector (entry D, Table 1) appeared to be promising at first, as the minimum concentration for transfection dropped even further to 150 μM, while maintaining low cytotoxicity. The efficiency was higher, but with a value of 1.8 % still left room for improvement. To further increase the distance between the DNA binding sites and additionally increase the solubility of the biotin label, biocytin was used, which led to vector **1** (entry E, Table 1). Maintaining a low cytotoxic profile, this final vector showed transfection at concentrations as low as 100 μM, whereas the maximum transfection efficiency (6.9 %) was achieved at a concentration of 150 μM. To rule out the possibility that the observed transfection can be solely attributed to the biotin moiety and no other changed characteristics (e. g., the additional positive charge of the biocytine moiety), control compound **2**, with an acetylated lysine (entry F, Table 1), was synthesized. The overall number of positive charges, both in vector **1** and control **2** is 6, hence the only difference is in the presence or absence of biotin. Nevertheless, **2** showed no signs of transfection at concentrations up to 600 μM. The toxicity of **2** was still low indicating that it might not be able to pass through the cell membrane. Based on these results, we set out to investigate the potential of the most promising vector **1** for cell‐specific gene transfection using **2** as a negative control.

First, we compared the transfection ability of **1** and **2** (150 μM) in HeLa and HEK293T cells. HeLa cells expose a high number of biotin receptors on their cellular surface (BR^+^), whereas 293T cells do not (BR^−^).^[16]^ The commercially available transfection agent polyethylenimine (PEI) was chosen for comparison as it forms polyplexes with DNA and also follows the endosomal uptake pathway,^[22]^ resembling the mode of action hypothesized for **1**. Just as reported for PEI, lysosomal degradation can possibly be avoided by the proton sponge effect.^[23]^ Commercially available Lipofectamine2000® on the other hand forms stable lipoplexes which use different means to escape the endosome.^[24]^

As illustrated in Figure 3, transfection vector **1** led to a decent expression of mCherry in HeLa cells whereas **2** did not appear to transport genetic material at all and PEI showed the highest transfection efficiency (Figure 3, left). In contrast to PEI, however, transfection efficiency of **1** was significantly reduced in 293T cells and remained low for the control vector **2** (Figure 3, right). Both facts that a) **1** revealed higher transfection efficiencies for the biotin‐receptor‐positive (BR^+^) HeLa cell line compared to the biotin‐receptor‐deficient (BR^−^) HEK293T cell line, and b) that the structurally similar, but biotin‐lacking control vector **2** was completely ineffective, point to a key role of the biotin moiety in mediating DNA uptake. However, a simple difference in the ability of the two vectors to condense DNA, which is of key importance for the passage through the cell membrane,^[25]^ could also be the reason for the observed difference in transfection behavior. Therefore, the size of the aggregates formed by plasmid DNA with **1** and **2** in solution was measured by DLS. Stock solutions of the peptides in DMSO were titrated to a solution of plasmid DNA. For both vectors a decrease in size was observed with increasing vector concentrations.

**Figure 3:**
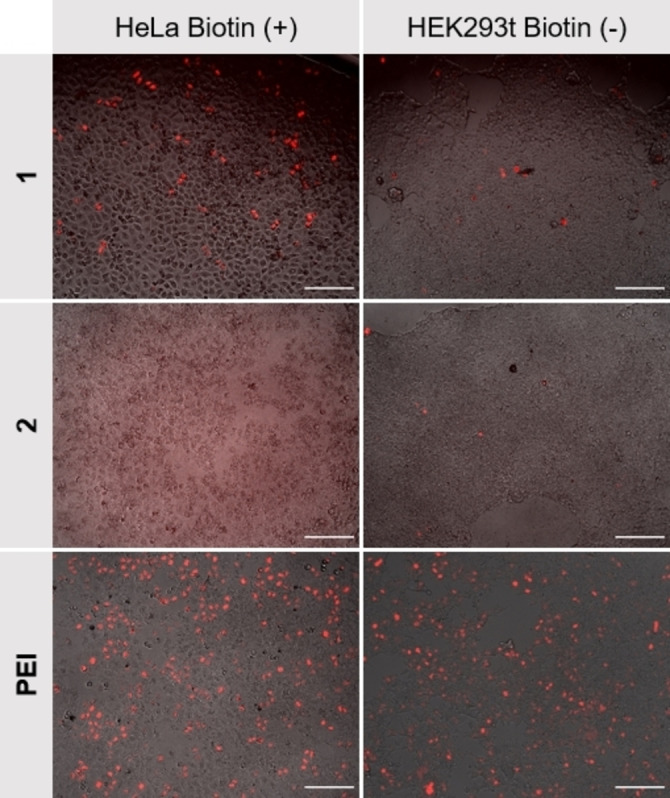
Example microscopy images of HeLa (left) and HEK293T (right) cells to compare transfection of pH 2B‐mCherry with **1** and **2** to commercially available linear PEI (25 kDa). Concentrations were: **1**, **2**=150 μM, pH 2B‐mCherry plasmid=1 μg mL^−1^. Scale bars: 200 μm; average transfection efficiencies were calculated by using CellProfiler®: HeLa‐**1**: 6.9%, HEK293T‐**1**: 0.7, HeLa‐**2**: 0 %, HEK293T‐**2**: 0.1 %, HeLa‐PEI: 28.5 %, HEK293T‐PEI: 39.8 %. At least five positions totalling 10 000 cells per combination of cell line and transfection reagent were analyzed.

In Figure 4A, the size distribution of both vectors is shown at a concentration of 150 μM (as previously used). Complex sizes for both peptides of 100 nm were observed. The spherical nature of aggregates formed by **1** with pH 2B‐mCherry DNA was confirmed by AFM (Figure 4B).^[26]^ Especially noteworthy is that the aggregates formed by **2** are of optimal size for transfection, while the slightly bigger aggregates formed by **1** are marginally above what is usually considered the optimal range.^[27]^ Thus, the ability to form small polyplexes does not seem to be the reason why **2** showed virtually no transfection. Hence, the hypothesis of the biotin receptor‐dependent uptake mechanism prevails and we set out to extend the study^[28]^ by three additional cell lines, including one BR^+^ cell line (A549) and two BR^−^ cell lines (CHO, HCT116).[^16^, ^29^] Again, the biotin‐lacking vector **2** was chosen as an all‐negative and PEI as an all‐positive control (for further controls, that is, Lipofectamine2000® and calcium phosphate nanoparticles see the Supporting Information). After staining cellular nuclei with Hoechst 33342 the percentage of transfected cells was calculated using CellProfiler®. The results are summarized in the heatmap shown in Figure 5.

**Figure 4:**
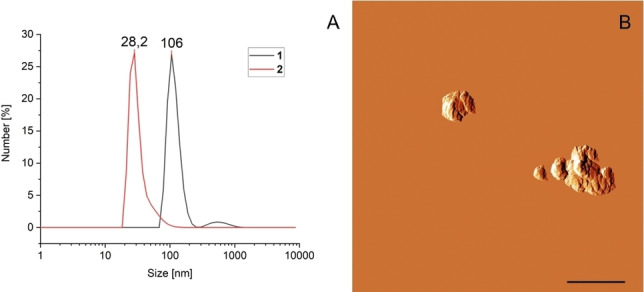
A) DLS measurements of substance **1** and **2** at a concentration of 150 μM with pH 2B‐mCherry (10 mg mL^−1^) in H_2_O; B) AFM‐image of **1** (125 μM) after incubation and spin‐coating with pH 2B‐mCherry (10 mg mL^−1^). Scale bar: 2 μm.

**Figure 5:**
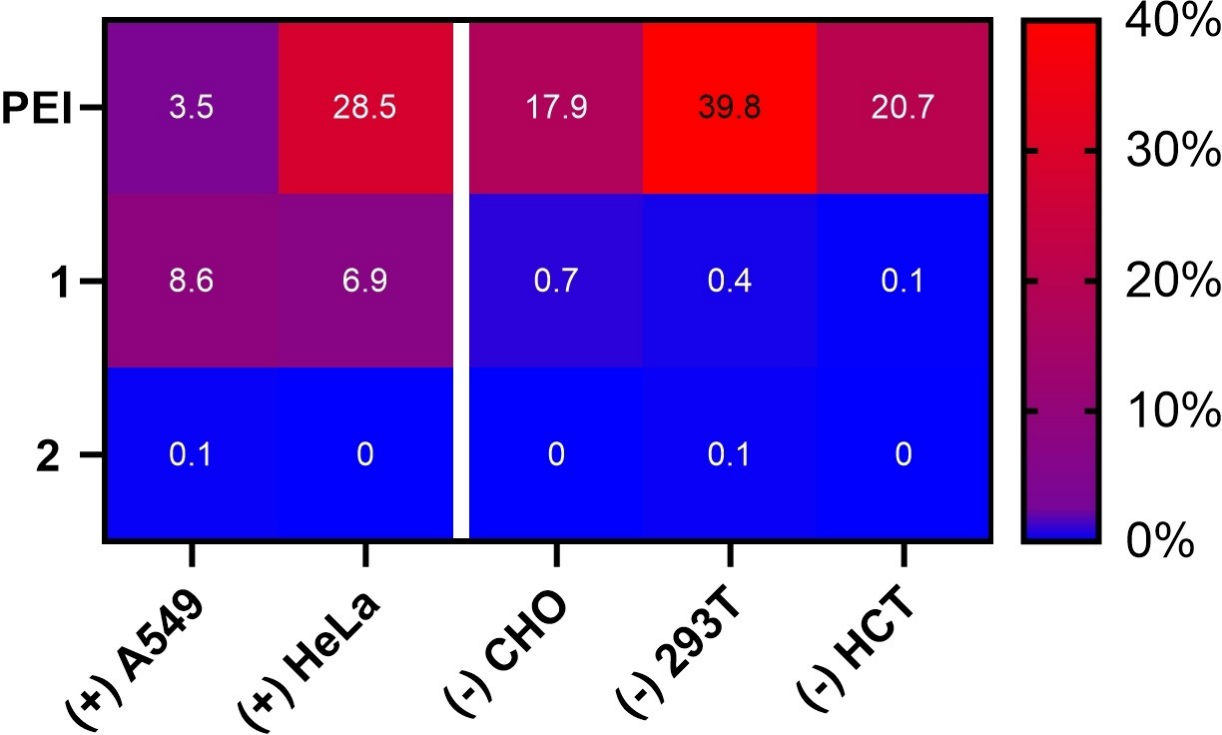
Heatmap representation of transfection efficiencies of **1**, **2** and linear PEI (25 kDa) on different cancer cell lines. (+) and (−) qualitatively describe the biotin–receptor density on the cellular surface. (Concentrations: **1** and **2**=150 μM, pH 2B‐mCherry plasmid=1 μg mL^−1^). Transfection efficiencies were calculated by employing a CellProfiler® pipeline that counted and calculated the fraction of H2B‐mCherry‐expressing cells out of the total number of cells stained with Hoechst 33342 (*N*=10 000).

The rows in Figure 5 represent the compounds employed, while the columns list the different cell types grouped according to their biotin receptor expression levels. Each tile is marked with a color gradient indicating transfection efficiency. Most notable is the difference between BR^+^ and BR^−^ cell lines. It is immediately apparent that **1** is a whole order of magnitude more efficient in terms of transfection for the two BR^+^ cell lines A549 and HeLa compared to the three BR^−^ cell lines CHO, 293T and HCT. On the other hand, the two controls PEI and **2** are almost indiscriminate in their ability/inability to transfect all cell lines. Although PEI does not seem to prefer any of the two groups of cell lines, its efficiency abates one order of magnitude for A549 cells. This is hardly surprising, as this cancer cell line is known for its resistance towards transfection.^[30]^ To our delight this BR^+^ cell line is transfected even more efficiently with the small‐molecule vector **1**.

Further evidence for a biotin receptor dependent uptake was obtained from competition experiments on HeLa cells. Addition of a fluorescein‐biotin conjugate reduced the transfection efficiency of **1** in a concentration dependent manner (Figure 6A), as the competing biotin derivative blocks the receptors on the cell surface. Furthermore, transfection was completely abolished by preincubation with Bafilomycin A1 (Figure 6B), which interferes with the endocytic pathway. Thus, the given data confirms a biotin receptor‐selective endocytic transfection mechanism for **1**.

**Figure 6:**
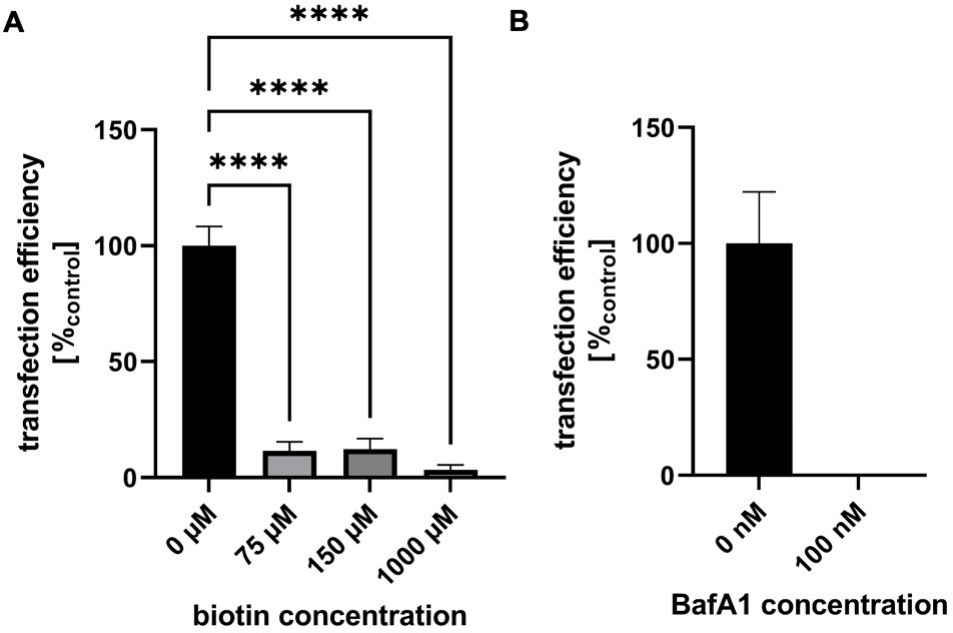
A) Biotin competition assay: HeLa cells were preincubated with fluorescein‐labeled biotin at the concentrations indicated. Transfection was performed by using vector **1** (300 μM in 100 μL culture growth medium) and 0.2 μg plasmid DNA. Transfection efficiency was measured by using Cell Profiler®. B) BafA1 effect assessment: HeLa cells were transfected by using the standard protocol **1** (150 μM in 100 μL culture growth medium) with and without preincubation with BafA1.

While efficiency and more importantly cell specificity were the pivotal aspects for the performance of our system, its toxicity is equally relevant when it comes to potential applications. While only having included HeLa cells in our initial screening, cell viability was probed for all tested cell lines in the presence of up to 200 μM of **1**. As shown in Figure 7 vector **1** is not cytotoxic in the concentration range necessary for transfection. A small decrease in cell viability was observed in CHO cells at a concentration of 200 μM, but at 150 μM, which is the concentration optimal for transfection, cell viability was still excellent. Additional toxicological data is shown in the Supporting Information.

**Figure 7:**
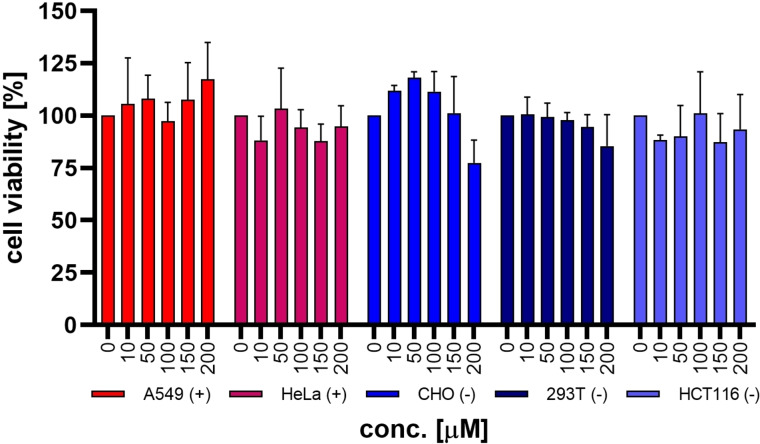
CellTiter 96® AQ_ueous_ One Solution Cell Proliferation Assay (Promega) performed according to the manufacturer's instructions on different cancer cell lines incubated with increasing concentrations of **1**. (+) and (−) qualitatively describe the biotin receptor density on the cellular surface.

To conclude, vector **1**, is a highly selective, nontoxic vector exhibiting a clear preference for two cell lines that express biotin receptors over three biotin receptor‐deficient cell lines. Vector **1** contains biocytin as receptor‐targeting moiety, connected to an array of potent anion binders via a flexible ethylene glycol linker, carrying a carefully optimized number of positively charged amino groups. Although both **1** and its biotin‐free relative **2** are able to condense DNA sufficiently, only **1** functions as an efficient transfection vector selective for cells expressing biotin receptors on their surface. To the best of our knowledge, this selectivity for BR^+^ over BR^−^ cell lines of approximately one order of magnitude is the highest currently reported. This makes the development of **1** an interesting showcase for how a small‐molecule approach based on the potent tetralysine‐GCP binding motif and a binding unit of similar size, allows the development of efficient targeting vectors.

## Conflict of interest

The authors declare no conflict of interest.

## Supporting information

As a service to our authors and readers, this journal provides supporting information supplied by the authors. Such materials are peer reviewed and may be re‐organized for online delivery, but are not copy‐edited or typeset. Technical support issues arising from supporting information (other than missing files) should be addressed to the authors.

Supporting Information

## Data Availability

The data that support the findings of this study are available in the supplementary material of this article.
