# Supplementary material for: Advances towards Cell‐Specific Gene Transfection: A Small‐Molecule Approach Allows Order‐of‐Magnitude Selectivity
Source: Chemistry. 2022 Jun 17;28(43):e202104618. doi: 10.1002/chem.202104618 (PMC9401007; doi:10.1002/chem.202104618)
Supplement: Supplementary file 1 — Supporting Information [file CHEM-28-0-s001.pdf]

# Chemistry–A European Journal

Supporting Information


## Advances towards Cell-Specific Gene Transfection: A Small-Molecule Approach Allows Order-of-Magnitude Selectivity


Thies Dirksmeyer, Paul Stahl, Cecilia Vallet, Shirley Knauer, Michael Giese, Carsten Schmuck, and Christoph Hirschhäuser*

# Table of Contents

1. General ... 2
2. Peptide synthesis ... 3
Synthesis of S1 ... 3
Synthesis of S2 ... 4
Synthesis of S3 ... 5
Synthesis of S4 ... 6
Synthesis of 1 ... 7
Synthesis of 2 ... 8
Synthesis of S5 ... 9
Synthesis of S6 ... 9
Synthesis of S7 ... 9
Synthesis of S8 ... 10
Synthesis of S9 ... 10
Synthesis of S10 ... 11
3. Physicochemical Experiments ... 12
3.1 Atomic Force Microscopy ... 12
3.2 Dynamic Light Scattering ... 12
4. Biological experiments ... 13
4.1 Toxicity ... 13
4.2 Transfection and Microscopy ... 14
4.3 Quantification of Transfection Results ... 16
4.4 Biotin Competition Assay and Bafilomycin A1 Experiment ... 17
5. Compound Characterization ... 18
HPLC and MS-spectra of S1 ... 18
HPLC and MS-spectra of S2 ... 19
HPLC and MS-spectra of S3 ... 20
HPLC and MS-spectra of S4 ... 21
NMR, HPLC and MS spectra of 1 ... 22
NMR, HPLC and MS spectra of 2 ... 24
NMR spectra of S9 ... 26
NMR spectra of S10 ... 27
Additional NMR spectra ... 28
References ... 30

## 1. General

All chemicals were used without further purification. Millipore water was obtained using a MicroPure system from TKA. Products were lyophilized using an Alpha 1-2 LD Plus from Christ.
All NMR spectra were either recorded at a DMX 300, Avance III HD 400 or Avance HD600 spectrometer from Bruker. All chemical shifts are reported as in parts per million (ppm) in relation to the deuterated solvent DMSO-$d_6$ or $CDCl_3$. The following abbreviation are used to describe the multiplicity of peaks: s: singlet, d: doublet, t: triplet, m: multiplet, br: broad. Coupling constants are given as J in Hz in relation to the spectrometers frequency and the distance between corresponding peaks.

Mass spectroscopy: All mass spectra were recorded using either amaZon SL (Bruker corporation) or Bruker maXis 4G (Bruker corporation). Products were dissolved in either methanol or DCM.

IR-Spectrometry: FT-IR spectra were recorded using FT-IR430 spectrometer (JASCO Germany GmbH)

Melting-points: Melting points were determined using B-540 (Buchi Labortechnik GmbH)

DLS: Dynamic light scattering was recorded using a Zetasizer-Nano ZS (Malvern Panalytical) and an incorporated HeNe Laser at a wavelength of 633 nm at an angle of 173°. Samples were measured in 100 µL PMMA cuvettes and recorded after two min. of equilibration time at 25 °C. Aliquots of **1** and **2** were added from a 1.5 mM stock solution in DMSO. DNA was dissolved in pure $H_2O$.

Atomic force microscopy (AFM) images were recorded using an InnovaNanoDrive AFM controller and an Innova atomic microscope (Veeco/Bruker) in semi-contact mode. N-doped silicon cantilevers (OMCL-AC160TS-R3; Olympus) were used as measuring needles. The solutions were spin coated on mica plates (Plano) for 60 min. Analytes were added after premixing DNA (5 µg/mL) with aliquots of a stock solution of **1** (1.5 mM) in DMSO. DNA was measured in pure water.

Determination of purity was achieved using RP-HPLC using UlitMate 3000 Pump (Dionex), UltiMate 3000 Column Compartment (Dionex), ASI-100 Automated Sample Injector (Dionex) and UVD340U UV detector (Dionex). Columns were used from YMC (YMC-Pack ODS-AQ or YMC-Triart C18, S-5 µM, 12 nm, 150 x 3.0 mm). Eluents MeOH+0.1% TFA and $H_2O$+0.1% TFA.

Preparative HPLC was used from SCPA comprised of an Azura P2.1L HPLC pump (Knauer), a TIDAS S500 MCS UV/NIR detector (J&M) and a LABOCOL Vario-4000 collector (Labomatic). A column from YMC (YMC-Actus ODS-AQ, S-5 µM, 12 nm, 150 x 20 mm) was used.

The synthesis of the guanidinocarbonylpyrrole building block was synthesized according to the literature.[1]

## 2. Peptide synthesis

### Synthesis of S1

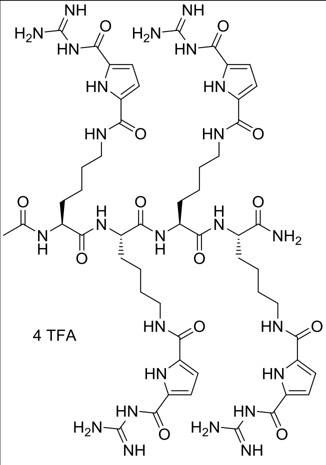


Chemical Formula: $C_{62}H_{81}F_{12}N_{25}O_{21}$
Molecular Weight: 1740,46

Solid-Phase peptide synthesis of **S1** was carried out with a CEM microwave apparatus in a 25.0 mL transparent polyethylene tube equipped with a sinter. Fmoc-Rink-Amide resin (200 mg, 0.320 mmol/g) was swollen in 10 mL of DCM/DMF (1:1) for 2 h. Initial Fmoc removal was achieved by addition of a 20% solution of piperidine in DMF (2x5 mL) and irradiation for 1 min and 5 min at 20 W with a maximum temperature of 60 °C. The resin was washed with DMF (6x5 mL). For the coupling of Fmoc-Lys(Alloc)-OH (58.0 mg, 0.128 mmol, 2.00 eq.), PyBOP (67.0 mg, 0.128 mmol, 2.00 eq.), DiPEA (44.0 µL, 0.256 mmol, 4.00 eq.) and DMF (5 mL) were added to the resin under Argon atmosphere and the mixture was irradiated by microwave at 20 W and a maximum temperature of 60 °C. This coupling was repeated to ensure full conversion. The resin was washed with DMF (3x5 mL). Fmoc-removal and coupling of Fmoc-Lys(Alloc)-OH for the second to forth amino acid was performed under the same conditions. The final Fmoc-group was removed and after consecutive washing with DMF (3x5 mL) the *N*-terminus was acetylated using acetic anhydride (58.0 µL, 0.610 mmol, 9.50 eq.) and DiPEA (109 µL, 0.640 mmol, 10.0 eq.) in DMF (5 mL) using microwave irradiation at 20 W for 10 min at a maximum temperature of 60 °C. The resin was washed with DMF (3x5 mL) The Alloc-protecting groups were removed by microwave irradiation with $Pd(PPh_3)_4$ (30.0 mg, 0.0256 mmol, 0.400 eq.) and $PhSiH_3$ (758 µL, 6.14 mmol, 96.0 eq.) in DCM (5 mL) at 20 W for 10 min at a maximum temperature of 30 °C. This step was repeated once. After this the resin was washed using DCM, DMF, 0.5% DiPEA/DMF, 0.2 M Diethyldithiocarbamate/DMF solution, MeOH, DCM and DMF (3x5 mL, 3 min). The coupling of the GCP unit was conducted using GCP(Boc)-OH*$NEt_3$ (204 mg, 0.512 mmol, 8.00 eq.), PyBOP (266 mg, 0.512 mmol, 8.00 eq.) and DiPEA (175 µL, 1.02 mmol, 16.0 eq.) in DMF (7 mL) with microwave irradiation at 20 W for 20 min and a maximum temperature of 60 °C. This step was repeated twice and controlled by Kaiser-Test. After that the resin was washed using DMF, MeOH and DCM (each 3x5 mL, 3 min) and dried under vacuum for 1 h. The resin was transferred to a round Schlenk flask equipped with a sinter and the peptide was cleaved over 1 h with a mixture of 10 mL TFA/TIS/$H_2O$ (95/2.5/2.5). This was repeated twice for 10 min each. The collected cleavage solutions were concentrated to about 2 mL under reduced pressure and the product was precipitated in 40 mL cold $Et_2O$. Afterwards the precipitate was centrifuged, washed again with 40 mL $Et_2O$ and subsequently dried by lyophilization. The product was isolated using RP-MPLC (30 – 50% MeOH+0.1% TFA) and obtained as a white solid (9.10 mg, 5.22 µmol, 8.2%).

**HRMS (ESI)**: m/z calcd. for $C_{54}H_{77}N_{25}O_{13}$+3H: 428.8784 [*M*+3H]$^{3+}$; found:428.8779

**Purity determined by RP-HPLC** (30 − 50% MeOH+0.1% TFA): 99%

**Melting point**: 259 °C (decomposition)

**Synthesis of S2**

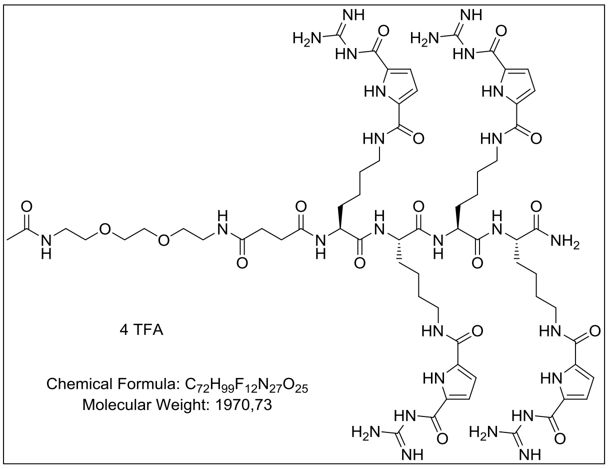


Solid-Phase peptide synthesis of **S2** was carried out with a CEM microwave apparatus in a 25.0 mL transparent polyethylene tube equipped with a sinter. Fmoc-Rink-Amide resin (400 mg, 0.320 mmol/g) was swollen in 10 mL of DCM/DMF (1:1) for 2 h. Initial Fmoc removal was achieved by addition of a 20% solution of piperidine in DMF (2x5 mL) and irradiation for 1 min and 5 min at 20 W with a maximum temperature of 60 °C. The resin was washed with DMF (6x5 mL). For the coupling of Fmoc-Lys(Alloc)-OH (116 mg, 0.256 mmol, 2.00 eq.), PyBOP (133 mg, 0.256 mmol, 2.00 eq.), DiPEA (87.4 µL, 0.512 mmol, 4.00 eq.) and DMF (7 mL) were added to the resin under Argon atmosphere and the mixture was irradiated by microwave at 20 W and a maximum temperature of 60 °C. This coupling was repeated to ensure full conversion. The resin was washed with DMF (3x5 mL). Fmoc-removal and coupling of Fmoc-Lys(Alloc)-OH for the second to forth amino acid was performed under the same conditions. The linker unit was coupled using **S10** (120 mg, 0,256 mmol, 2.00 eq.), PyBOP (133 mg, 0.256 mmol, 2.00 eq.), DiPEA (87.4 µL, 0.512 mmol, 4.00 eq.) in DMF (7 mL) microwave irradiation at 20 W and a maximum temperature of 60 °C. The final Fmoc-group was removed and after consecutive washing with DMF (3x5 mL) the *N*-terminus was acetylated using acetic anhydride (115 µL, 1.22 mmol, 9.50 eq.) and DiPEA (219 µL, 1.28 mmol, 10.0 eq.) in DMF (7 mL) using microwave irradiation at 20 W for 10 min at a maximum temperature of 60 °C. The resin was washed with DMF (3x5 mL) The Alloc-protecting groups were removed by microwave irradiation with $Pd(PPh_3)_4$ (59.2 mg, 0.0512 mmol, 0.400 eq.) and $PhSiH_3$ (1.52 mL, 12.3 mmol, 96.0 eq.) in DCM (6 mL) at 20 W for 10 min at a maximum temperature of 30 °C. This step was repeated once. After this the resin was washed using DCM, DMF, 0.5% DiPEA/DMF, 0.2 M Diethyldithiocarbamate/DMF solution, MeOH, DCM and DMF (3x5 mL, 3 min). The coupling of the GCP unit was conducted using GCP(Boc)-OH*$NEt_3$ (407 mg, 1.02 mmol, 8.00 eq.), PyBOP (533 mg, 1.02 mmol, 8.00 eq.) and DiPEA (350 µL, 2.05 mmol, 16.0 eq.) in DMF (10 mL) with microwave irradiation at 20 W for 20 min and a maximum temperature of 60 °C. This step was repeated twice and controlled by Kaiser-Test. After that the resin was washed using DMF, MeOH and DCM (each 3x5 mL, 3 min) and dried under vacuum for 1 h. The resin was transferred to a round schlenk flask equipped with a frit and the peptide was cleaved over 1 h with a mixture of 10 mL TFA/TIS/$H_2O$ (95/2.5/2.5). This was repeated twice for 10 min each. The collected cleavage solutions were evaporated to about 2 mL under reduced pressure and the product was precipitated in 40 mL cold $Et_2O$. Afterwards the precipitate was centrifuged, washed with 40 mL $Et_2O$ again and subsequently dried by lyophilization. The product was isolated using RP-MPLC (30 – 50 MeOH+0.1% TFA) and obtained as a white solid (5.20 mg, 2.64 µmol, 2.1%).

**HRMS (ESI)**: m/z calcd. for $C_{64}H_{95}N_{27}O_{17}$: 505.5873 $[M+3H]^{3+}$; found:505.5874

**Purity determined by RP-HPLC** (30 − 50% MeOH+0.1% TFA): 94%

**Melting point**: 230 °C (decomposition)

**Synthesis of S3**

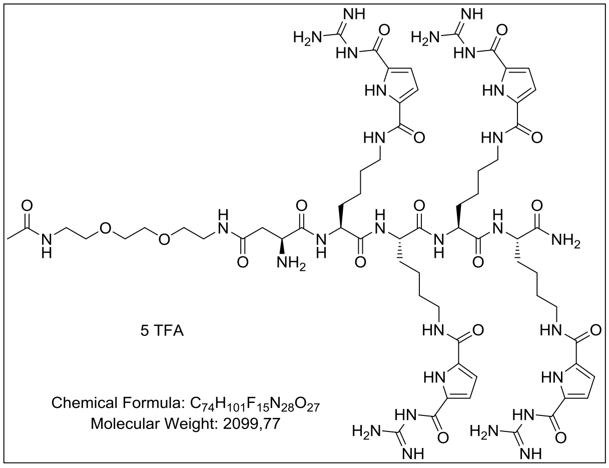


Solid-Phase peptide synthesis of **S3** was carried out with a CEM microwave apparatus in a 25.0 mL transparent polyethylene tube equipped with a sinter. Fmoc-Rink-Amide resin (200 mg, 0.320 mmol/g) was swollen in 10 mL of DCM/DMF (1:1) for 2 h. Initial Fmoc removal was achieved by addition of a 20% solution of piperidine in DMF (2x5 mL) and irradiation for 1 min and 5 min at 20 W with a maximum temperature of 60 °C. The resin was washed with DMF (6x5 mL). For the coupling of Fmoc-Lys(Alloc)-OH (58.0 mg, 0.128 mmol, 2.00 eq.), PyBOP (67.0 mg, 0.128 mmol, 2.00 eq.), DiPEA (44.0 µL, 0.256 mmol, 4.00 eq.) and DMF (5 mL) were added to the resin under Argon atmosphere and the mixture was irradiated by microwave at 20 W and a maximum temperature of 60 °C. This coupling was repeated to ensure full conversion. The resin was washed with DMF (3x5 mL). Fmoc-removal and coupling of Fmoc-Lys(Alloc)-OH for the second to forth amino acid was performed under the same conditions. The linker unit was coupled using **S10** (75.0 mg, 0,128 mmol, 2.00 eq.), PyBOP (67.0 mg, 0.128 mmol, 2.00 eq.), DiPEA (44.0 µL, 0.256 mmol, 4.00 eq.) in DMF (5 mL) under microwave irradiation at 20 W and a maximum temperature of 60 °C. The final Fmoc-group was removed and after consecutive washing with DMF (3x5 mL) the *N*-terminus was acetylated using acetic anhydride (58.0 µL, 0.610 mmol, 9.50 eq.) and DiPEA (109 µL, 0.640 mmol, 10.0 eq.) in DMF (5 mL) using microwave irradiation at 20 W for 10 min at a maximum temperature of 60 °C. The resin was washed with DMF (3x5 mL). The Alloc-protecting groups were removed by microwave irradiation with $Pd(PPh_3)_4$ (30.0 mg, 0.0256 mmol, 0.400 eq.) and $PhSiH_3$ (758 µL, 6.14 mmol, 96.0 eq.) in DCM (5 mL) at 20 W for 10 min at a maximum temperature of 30 °C. This step was repeated once. After this the resin was washed using DCM, DMF, 0.5% DiPEA/DMF, 0.2 M Diethyldithiocarbamate/DMF solution, MeOH, DCM and DMF (3x5 mL, 3 min). The coupling of the GCP unit was conducted using GCP(Boc)-OH*$NEt_3$ (204 mg, 0.512 mmol, 8.00 eq.), PyBOP (266 mg, 0.512 mmol, 8.00 eq.) and DiPEA (175 µL, 1.02 mmol, 16.0 eq.) in DMF (7 mL) with microwave irradiation at 20 W for 20 min and a maximum temperature of 60 °C. This step was repeated twice and controlled by Kaiser-Test. After that the resin was washed using DMF, MeOH and DCM (each 3x5 mL, 3 min) and dried under vacuum for 1 h. The resin was transferred to a round schlenk flask equipped with a frit and the peptide was cleaved over 1 h with a mixture of 10 mL TFA/TIS/$H_2O$ (95/2.5/2.5). This was repeated twice for 10 min each. The collected cleavage solutions were evaporated to about 2 mL under reduced pressure and the product was precipitated in 40 mL cold $Et_2O$. Afterwards the precipitate was centrifuged, washed with 40 mL $Et_2O$ again and subsequently dried by lyophilization. The product was isolated using RP-MPLC (30 – 50 MeOH+0.1% TFA) and obtained as a white solid (7.80 mg, 3.72 µmol, 5.8%).

**HRMS (ESI)**: m/z calcd. for $C_{64}H_{96}N_{28}O_{17}$ 383.1950 $[M+4H]^{4+}$; found:383.1947

**Purity determined by RP-HPLC** (30 − 50% MeOH+0.1% TFA): 93%

**Melting point**: 230 °C (decomposition)

**Synthesis of S4**

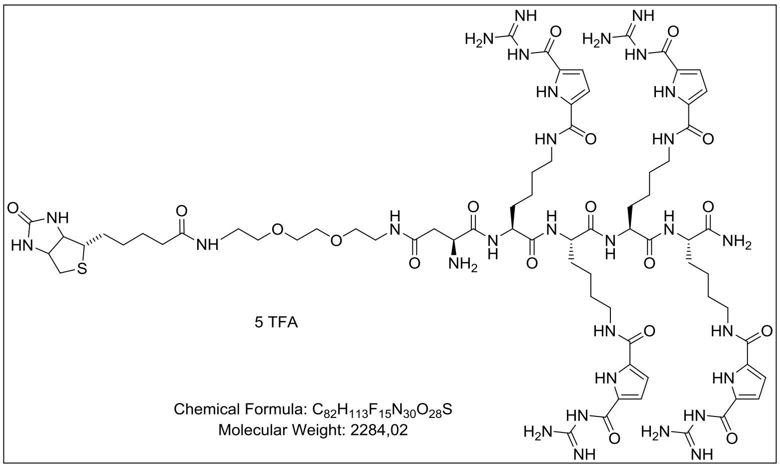


Solid-Phase peptide synthesis of **S4** was carried out in a round Schlenk flask equipped with a sinter. Fmoc-Rink-Amide resin (100 mg, 0.5 mmol/g) was swollen in 10 mL of DCM/DMF (1:1) for 2 h. Initial Fmoc removal was achieved by addition of a 20% solution of piperidine in DMF (2x5 mL) and shaking for 20 min under argon. The resin was washed with DMF (3x5 mL). For the coupling of Fmoc-Lys(Alloc)-OH (67.9 mg, 0.150 mmol, 3.00 eq.), PyBOP (78.1 mg, 0.150 mmol, 3.00 eq.), DiPEA (51.2 µL, 0.300 mmol, 6.00 eq.) and DMF (5 mL) were added under an atmosphere of argon to the resin and the mixture was shaken for 1 h. The resin was washed with DMF (3x5 mL). Fmoc-removal and coupling of Fmoc-Lys(Alloc)-OH for the second to forth amino acid was performed under the same conditions. The linker unit **S10** (87.8 mg, 0,150 mmol, 3.00 eq.) was coupled with PyBOP (78.1 mg, 0.150 mmol, 3.00 eq.) and DiPEA (51.2 µL, 0.150 mmol, 3.00 Äquiv.) for 1 h at rt. Following this the Fmoc group was removed with the aforementioned solution (2x5 mL) for 20 min. Biotin (36.6 mg, 0.150 mmol, 3.00 eq.) was coupled for 1 h using PyBOP (78.1 mg, 0.150 mmol. 3.00 eq.) and DiPEA (48.5 µL, 0.300 mmol, 6.00 eq.) in DMF (6 mL) while shaking. ***All subsequent steps were conducted under Argon in order to prevent oxidation***.* The Alloc-protecting groups were removed using $Pd(PPh_3)_4$ (28.9 mg, 0.025 mmol, 0.500 eq.), $PhSiH_3$ (770 µL, 6.00 mmol, 120 eq.) in DCM (2x6 mL, 20 mins) while shaking. Afterwards the resin was washed using DCM, DMF, 0.5% DiPEA/DMF, 0.2 M Diethyldithiocarbamate/DMF solution, MeOH, DCM and DMF (3x5 mL 3 min). Coupling of GCP(Boc)-OH*$NEt_3$ (239 mg, 0.60 mmol, 12.0 eq.), PyBOP (312 mg, 0.60 mmol, 12.0 eq.) and DiPEA (295 µL, 1.80 mmol, 36.0 eq.) in DMF (7 mL) was performed in 1 h. This step was repeated twice and monitored by Kaiser-Test. Following this the resin was washed with DMF, MeOH and DCM (each 3x5 mL) and dried under reduced pressure for 1 h. The peptide was cleaved off the resin using a 10 mL cleavage cocktail of TFA/TIS/$H_2O$ (95:2.5:2.5) for 1 h. This was repeated twice for 10 min each. The collected cleavage solutions were evaporated to about 2 mL and flushed with argon. The peptide was precipitated in 40 mL of cold $Et_2O$, centrifuged and washed with 40 mL $Et_2O$ again. The crude product was dried using lyophilization. The product was isolated using preparative RP-HPLC (30 – 50 MeOH+0.1% TFA) and obtained as a white solid (10.3 mg, 4.51 µmol, 9.0%).

**HRMS (ESI)**: m/z calcd. for $C_{72}H_{108}N_{30}O_{18}S$ 429.2117 [$M$+4H]$^{4+}$; found:429.2123

**Purity determined by RP-HPLC** (30 − 50% MeOH+0.1% TFA): 96%

**Melting point**: 170 °C (decomposition)

***Comment:** *The biotin sulfide moiety is oxidized if contact with air is allowed for a prolonged time. This was observed during the synthesis of related compounds. Sample NMR – spectra of an isolated sulfoxide are shown on page 27.*

**Synthesis of 1**

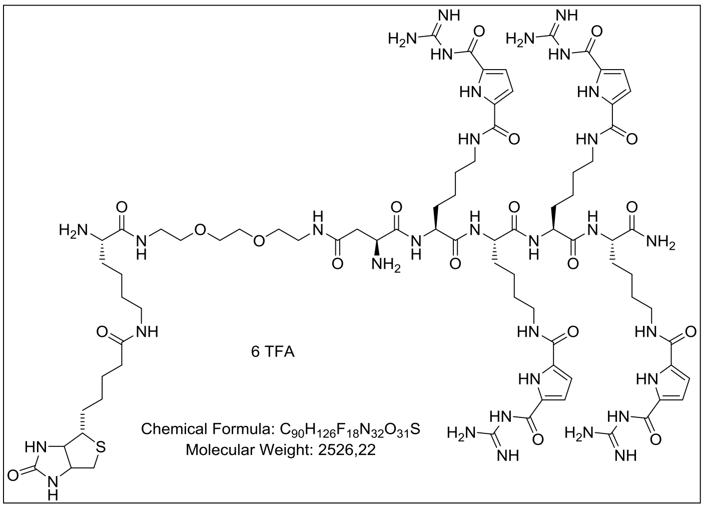


Solid-Phase peptide synthesis of **1** was carried out in a round Schlenk flask equipped with a frit. Fmoc-Rink-Amide resin (200 mg, 0.360 mmol/g) was swollen in 10 mL of DCM/DMF (1:1) for 2 h. Initial Fmoc removal was achieved by addition of a 20% solution of piperidine in DMF (2x5 mL) and shaking for 20 min under Argon. The resin was washed with DMF (3x5 mL). For the coupling of Fmoc-Lys(Alloc)-OH (98.0 mg, 0.216 mmol, 3.00 eq.), HCTU (89.0 mg, 0.216 mmol, 3.00 eq.), DiPEA (74.0 µL, 0.432 mmol, 6.00 eq.) and DMF (5 mL) were added under an atmosphere of argon to the resin and shaken for 1 h. The resin was washed with DMF (3x5 mL). Fmoc-removal and coupling of Fmoc-Lys(Alloc)-OH for the second to forth amino acid was performed under the same conditions. The linker unit **S10** (126 mg, 0,216 mmol, 3.00 eq.) was coupled with HCTU (89.0 mg, 0.216 mmol, 3.00 eq.) and DiPEA (74.0 µL, 0.432 mmol, 6.00 eq.) for 1 h while shaking. After consecutive Fmoc-removal and washing with DMF (3x5 mL), Boc-Lys(Fmoc)-OH (101 mg, 0.216 mg, 3.00 eq.) was coupled using HCTU (89.0 mg, 0.216 mmol, 3.00 eq.) and DiPEA (74.0 µL, 0.432 mmol, 6.00 eq.) for 1 h. Following this the Fmoc group was removed with the aforementioned solution (2x5 mL) for 20 min. Biotin (53.0 mg, 0.216 mmol, 3.00 eq.) was coupled for 1 h using PyBOP (112 mg, 0.216 mmol. 3.00 eq.) and DiPEA (74.0 µL, 0.432 mmol, 6.00 eq.) in DMF (6 mL) while shaking. ***All subsequent steps were conducted under Argon in order to prevent oxidation***.* The Alloc-protecting groups were removed using $Pd(PPh_3)_4$ (42.0 mg, 0.0360 mmol, 0.500 eq.), $PhSiH_3$ (1.10 mL, 8.60 mmol, 120 eq.) in DCM (2x6 mL, 20 mins) while shaking. Afterwards the resin was washed using DCM, DMF, 0.5% DiPEA/DMF, 0.2 M Diethyldithiocarbamate/DMF solution, MeOH, DCM and DMF (3x5 mL 3 min). Coupling of GCP(Boc)-OH*$NEt_3$ (343 mg, 0.768 mmol, 12.0 eq.), PyBOP (450 mg, 0.768 mmol, 12.0 eq.) and DiPEA (393 µL, 1.54 mmol, 36.0 eq.) in DMF (7 mL) was performed in 1 h. This step was repeated twice and monitored by Kaiser-Test. Following this the resin was washed with DMF, MeOH and DCM (each 3x5 mL) and dried under reduced pressure for 1 h. The peptide was cleaved off the resin using a 10 mL cleavage cocktail of TFA/TIS/$H_2O$ (95:2.5:2.5) for 1 h. This was repeated two times for 10 min each. The collected cleavage solutions were evaporated to about 2 mL and flushed with Argon. The peptide was precipitated in 40 mL of cold $Et_2O$, centrifuged and washed with 40 mL $Et_2O$ again. The crude product was dried using lyophilization. The product was isolated using preparative RP-HPLC (36% MeOH+0.1% TFA) and obtained as a white solid (6.58 mg, 2.60 µmol, 3,6%).

**$^1$H-NMR** (600 MHz, DMSO-$d_6$): δ/ppm: 12.25 (s, 4H), 11.34 (s, 4H), 7.75-8.59 (m, 27H), 7.32 (s, 2H), 7.19 (s, 1H), 7.04-7.10 (m, 8H), 6.81 (br.s, 4H, pyrrole-C*H*), 6.54 (s, 2H), 6.42 (s, 1H, biotin-N*H*), 6.39 (s, 1H, biotin-N*H*), 5.32-5.33 (m, 1H), 4.13-4.31 (m, 7H), 3.70 (t, *J* = 6.30 Hz, 1H), 3.59 (m, 1H), 3.50 (br. s, 3H), 3.42 (m, 3H),** 3.19-3.20 (m, 6H), 3.07-3.10 (m, 2H), 2.99-3.00 (m, 2H), 2.77 (dd, *J* = 4.92 Hz, 12.51 Hz, 1H, biotin-C*H*), 2.73-2.75 (m, 2H), 2.54 (d, *J* = 12.51 Hz, 1H, biotin-C*H*), 2.52 (br. s, 2H), 1.97-2.05 (m, 4H), 1.23-1.68 (m, 30H).

**$^{13}$C-NMR** (150 MHz, DMSO-$d_6$): δ/ppm **=** 173.5, 171.9, 171.6, 171.2, 168.7, 168.6, 168.2, 162.7 (biotin-N-(C=O)-N), 159.0, 158.5, 158.3, 158.1, 158.0, 157.9, 132.4-132.5 (pyrrol-$C_{quart}$), 125.5-125.8***, 120.2, 118.2, 116.2, 115.0-115.2, 114.3, 112.3, 69.5, 68.8, 68.8 (Glycol-$CH_2$), 61.0 (biotin-bridgehead), 59.2 (biotin-bridgehead), 55.4 (biotin-CH), 53.1, 53.0, 52.8, 52.6, 52.3, 52.1, 49.1, 40.0, 38.7, 38.1, 35.7 ($CH_2$), 35.1-35.2 ($CH_2$), 34.4 ($CH_2$), 31.2-31.9 ($CH_2$) 30.9 ($CH_2$), 28.7-28.9 ($CH_2$), 28.2 (biotin-$CH_2$), 28.0 (biotin-$CH_2$), 25.3, 23.1 ($CH_2$), 22.7-22.9 ($CH_2$), 21.6 ($CH_2$).

*Assignments are given only for those signals, which could be unambiguously identified 2D NMR (COSY, HSQC, HMBC) data and comparison with reference spectra.*[2]

**HRMS (ESI)**: m/z calcd. for $C_{78}H_{120}N_{32}O_{19}S$ 461.2355 [*M*+4H]$^{4+}$; found:461.2354

**Purity determined by RP-HPLC** (30 − 50% MeOH+0.1% TFA): 99%

**Melting point**: 160 °C (decomposition)

***Comment:** *The biotin sulfide moiety is oxidized if contact with air is allowed for a prolonged time. This was observed during the synthesis of related compounds. Sample NMR – spectra of an isolated sulfoxide are shown on page 27.*

****Comment:** *Further resonances hidden by the solvent signals.*

*****Comment:** *These resonances could only be identified after application of a Lorenzian-Gaussian window function and were confirmed by comparison to reference spectra.*

**Synthesis of 2**

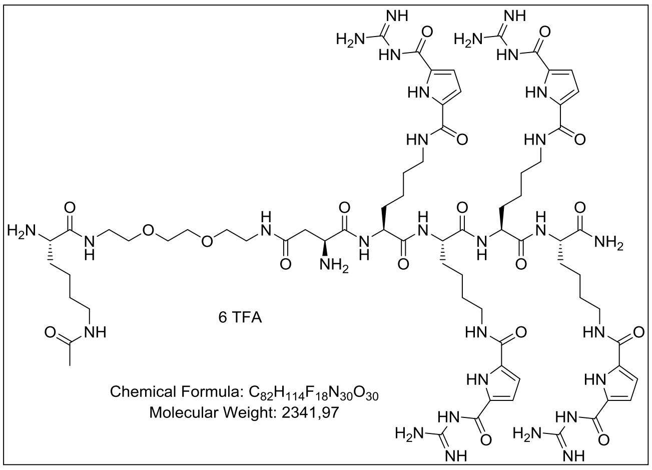


Solid-Phase peptide synthesis of **2** was carried out with a CEM microwave apparatus in a 25.0 mL transparent polyethylene tube equipped with a sinter. Fmoc-Rink-Amide resin (300 mg, 0.360 mmol/g) was swollen in 10 mL of DCM/DMF (1:1) for 2 h. Initial Fmoc removal was achieved by addition of a 20% solution of piperidine in DMF (2x5 mL) and irradiation for 1 min and 5 min at 20 W with a maximum temperature of 60 °C. The resin was washed with DMF (6x5 mL). For the coupling of Fmoc-Lys(Alloc)-OH (204 mg, 0.450 mmol, 3.00 eq.), HCTU (186 mg, 0.450 mmol, 3.00 eq.), DiPEA (154 µL, 0.900 mmol, 6.00 eq.) and DMF (7 mL) were added to the resin under an atmosphere of Argon and the mixture was irradiated by microwave at 20 W and a maximum temperature of 60 °C. This coupling was repeated to ensure full conversion. The resin was washed with DMF (3x5 mL). Fmoc-removal and coupling of Fmoc-Lys(Alloc)-OH for the second to forth amino acid was performed under the same conditions. The linker unit was coupled using **S10** (263 mg, 0,450 mmol, 3.00 eq.), HCTU (186 mg, 0.450 mmol, 3.00 eq.), DiPEA (154 µL, 0.900 mmol, 6.00 eq.) in DMF (7 mL) under microwave irradiation at 20 W and a maximum temperature of 60 °C for 20 min. Boc-Lys(Fmoc)-OH (211 mg, 0.450 mg, 3.00 eq.) was coupled using HCTU (186 mg, 0.450 mmol, 3.00 eq.) and DiPEA (154 µL, 0.900 mmol, 6.00 eq.) for 30 min at previously mentioned elevated temperatures. The final Fmoc-group was removed and after consecutive washing with DMF (3x5 mL) the *N*-terminus was acetylated using acetic anhydride (135 µL, 1.42 mmol, 9.50 eq.) and DiPEA (256 µL, 150 mmol, 10.0 eq.) in DMF (7 mL) using microwave irradiation at 20 W for 10 min at a maximum temperature of 60 °C. The resin was washed with DMF (3x5 mL) The Alloc-protecting groups were removed by microwave irradiation with $Pd(PPh_3)_4$ (86.7 mg, 0.0750 mmol, 0.500 eq.) and $PhSiH_3$ (2.22 mL, 18 mmol, 120 eq.) in DCM (8 mL) at 20 W for 10 min at a maximum temperature of 30 °C. This step was repeated once. After this the resin was washed using DCM, DMF, 0.5% DiPEA/DMF, 0.2 M Diethyldithiocarbamate/DMF solution, MeOH, DCM and DMF (3x5 mL, 3 min). The coupling of the GCP unit was conducted using GCP(Boc)-OH*$NEt_3$ (715 mg, 1.20 mmol, 8.00 eq.), PyBOP (937 mg, 1.20 mmol, 8.00 eq.) and DiPEA (820 µL, 4.80 mmol, 32.0 eq.) in DMF (10 mL) with microwave irradiation at 20 W for 30 min and a maximum temperature of 60 °C. This step was repeated twice and controlled by Kaiser-Test. After that the resin was washed using DMF, MeOH and DCM (each 3x5 mL, 3 min) and dried under vacuum for 1 h. The resin was transferred to a round Schlenk flask equipped with a sinter and the peptide was cleaved over 1 h with a mixture of 10 mL TFA/TIS/$H_2O$ (95/2.5/2.5). This was repeated twice for 10 min each. The collected cleavage solutions were evaporated to about 2 mL under reduced pressure and the product was precipitated in 40 mL cold $Et_2O$. Afterwards the precipitate was centrifuged, washed with 40 mL $Et_2O$ again and subsequently dried by lyophilization. The product was isolated using RP-MPLC (30 – 50 MeOH+0.1% TFA) and obtained as a white solid (7.80 mg, 3.33 µmol, 2.2%).

**$^1$H-NMR** (600 MHz, DMSO-$d_6$): δ/ppm = 8.59-8.58 (d, *J* = 7.84 Hz, 2H, amide-N*H*), 8.51-8.50 (t, *J* = 5.20 Hz, amide-N*H*), 8.41 (br. s, 6H, guanidine-N*H*), 8.35-8.33 (t, *J* = 5.30 Hz, 3H, amide-N*H*), 8.18-8.17 (d, *J* = 7.84 Hz, 2H, amide-N*H*), 7.87-7.85 (d, *J* = 7.84 Hz, 1H, amide-N*H*), 7.81-7.78 (m, 3H, amide-N*H)*, 7.32 (br. s, 1H, amide-N*H*), 7.03 (m, 4H, pyrrole-C*H*), 6.82 (br. s, 4H, pyrrole-C*H*), 4.27-4.15 (m, 6H, alpha-C*H*), 3.71-3.69 (t, *J* = 6.42 Hz, 1H), 3.49 (br. s, 4H), 3.42-3.41 (m, 4H), 3.21 (m, 12H, glycol-C*H*, lys-C*H*), 3.00-2.97 (q, *J* = 6.48 Hz, 3H), 2.75-2.72 (m, 1H, C*H*), 1.78 (br. s, 3H, acetyl-C$H_3$), 1.67 (m, 8H, lys-C*H*), 1.54-1.48 (m, 16H, lys-C*H*), 1.38-1.22 (m, 22H, lys-C*H*).

**$^{13}$C-NMR** (150 MHz, DMSO-$d_6$): δ/ppm = 173.48 ($C_q$), 171.24 ($C_q$), 169.08 ($C_q$), 168.68 ($C_q$), 168.61 ($C_q$), 159.12 ($C_q$), 158.53-157.91 (q, $J_{CF}$ = 30.5 Hz, $CF_3CO_2$), 120.23-114.25 (q, $J_{CF}$ = 307 Hz, *C*$F_3CO_2$), 112.28 (pyrrole-*C*), 69.49 (*C*$H_2$), 69.47 (*C*$H_2$), 68.86 (*C*$H_2$), 52.94 (α-*C*H), 52.83 (α-*C*H), 52.55 (α -*C*H), 52.29 (α -*C*H), 52.16 (α-*C*H), 49.11 (alpha-*C*H), 40.06 (glycole-*C*H), 39.91 (glycole-*C*H), 38.75 (*C*$H_2$), 38.19 (*C*$H_2$), 31.64 (lys-*C*$H_2$), 30.83 (lys-*C*$H_2$), 28.85 (lys-*C*$H_2$), 28.70 (lys-*C*$H_2$), 23.07 (lys-*C*$H_2$), 22.75 (acetyl-*C*$H_3$), 21.57 (lys-*C*$H_2$).
Assignments based on $^1$H NMR, $^{13}$C NMR (DEPT-Q) and 2D NMR (COSY, HSQC, HMBC) experiments.

**HRMS (ESI)**: m/z calcd. for $C_{70}H_{108}N_{30}O_{18}$ 415.2198 [*M*+4H]$^{4+}$; found:461.2187

**Purity determined by RP-HPLC** (30 − 50% MeOH+0.1% TFA): 93%

**Melting point**: 156 °C (decomposition)

**Synthesis of S5**

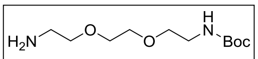


Chemical Formula: C11H24N2O4
Molecular Weight: 248,32

Following a literature known procedure[3] 2,2'-(ethylenedioxy)bis(ethylamine) (7.09 mL, 48.3 mmol, 6.00 eq.) was dissolved in DCM (41 mL) and di-*tert*-butyldicarbonate (1.76 g, 8.05 mmol, 1.00 eq.) in DCM (16 mL) was added with a precision addition funnel over 2 h at 0 °C. The reaction mixture was then stirred for 18 h at rt. The solvent was removed under reduced pressure and the colorless oil was taken up in $H_2O$ (16 mL). The aqueous phase was extracted with DCM (4x20 mL). The combined organic phases were dried over $MgSO_4$ filtered and concentrated *in vacuo*. The product was obtained as a colorless oil (2.04 mg, 8.20 mmol, quant.).

**$^1$H-NMR** (300 MHz, $CDCl_3$): δ/ppm = 5.15 (s, 1H), 3.61 (s, 4H), 3.55-3.50 (m, 4H), 3.32-3.27 (t, *J* = 5.00 Hz, 2H), 2.90-2.87 (t, *J* = 5.00 Hz, 2H), 1.43 (s, 9H).

**$^{13}$C-NMR** (100 MHz, $CDCl_3$): δ/ppm = 156.15, 79.29, 73.26, 70.33, 41.74, 40.45, 28.53.

*The spectroscopic data was consistent with that reported in the literature.*[3]

**Synthesis of S6**

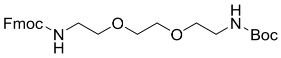


Chemical Formula: C26H34N2O6
Molecular Weight: 470,57

Following a literature known procedure [4] a solution of **S5** (1.00 g, 4.00 mmol, 1.00 eq.) in THF (20 mL) was added to a solution of $K_2CO_3$ (2.80 g) in $H_2O$ (20 mL). After that Fmoc-Cl (1.14 g, 4.40 mmol, 1.10 eq.) was added and the reaction mixture was stirred for 18 h at rt. Afterwards THF was evaporated under reduced pressure and the remaining aqueous phase was extracted with EtOAc (3x20 mL). The combined organic phases were dried over $Na_2SO_4$, filtered and the solvent removed under reduced pressure to yield the product **S6** (1.80 g, 4.00 mmol, quant.) as a colorless oil.

**$^1$H-NMR** (300 MHz, $CDCl_3$): δ/ppm = 7.78-7.75 (d, *J* = 7.30 Hz, 2H), 7.61-7.59 (d, *J* = 7.30 Hz, 2H), 7.42-7.38 (t, *J* = 7.50 Hz, 2H), 7.34-7.28 (td, *J* = 7.40, 1.20 Hz, 2H), 4.42-4.40 (d, *J* = 7.00 Hz, 2H), 4.25-4.21 (t, *J* = 7.00 Hz, 1H), 3.62-3.52 (m, 8H), 3.41 (br. s, 2H), 3.33-3.29 (t, *J* = 4.90 Hz, 2H), 1.43 (s, 9H).

**$^{13}$C-NMR** (100 MHz, $CDCl_3$): 156.59, 156.09, 144.07, 141.40, 128.01, 127.13, 125.16, 120.05, 70.35, 70.28, 66.70, 65.24, 47.36, 41.03, 28.50.

*The spectroscopic data was consistent with that reported in the literature.*[4]

**Synthesis of S7**

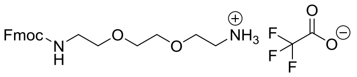


Chemical Formula: C23H27F3N2O6
Molecular Weight: 484,47

Following a literature known procedure[4] **S6** (1.80 g, 4.00 mmol, 1.00 eq.) was dissolved in DCM (23 mL). TFA (10 mL) was added and the reaction was stirred for 30 min at rt. Afterwards the solvents were removed under reduced pressure and coevaporated with toluene (2x15 mL). The crude product was dissolved in DCM (5 mL) and precipitated in $Et_2O$ overnight. The white precipitate was filtered off and dried *in vacuo* to yield the product **S7** (1.00 g, 2.80 mmol, 70%) as a white solid.

**$^1$H-NMR** (300 MHz, DMSO $d_6$): δ/ppm = 7.84-7.81 (d, *J* = 7.20 Hz, 2H), 7.68-7.66 (d, *J* = 7.40 Hz, 2H), 7.45-7-41 (t, *J* = 7.40 Hz, 2H), 7.36-7.31 (td, *J* = 7.40, 1.30 Hz, 2H), 4.40-3.38 (d, *J* = 6.80 Hz, 2H), 4.25-4.21 (t, *J* = 6.80 Hz, 1H), 3.76-3.67 (m, 8H), 3.17-3.14 (m, 2H), 3.12-3.08 (m, 2H).

**$^{13}$C-NMR** (100 MHz, $CDCl_3$): δ/ppm = 158.04, 156.21, 143.90, 140.76, 127.62, 127.06, 125.14, 120.15, 69.66, 69.39, 69.09, 66.67, 65.31, 46.72, 40.05, 38.61.

*The spectroscopic data was consistent with that reported in the literature.*[4]

**Synthesis of S8**

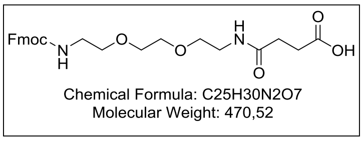


Following a literature known procedure[4] **S7** (1.00 g, 2.80 mmol, 1.00 Eq) and succinic anhydride (311 mg, 3.10 mmol, 1.10 eq.) were dissolved in DCM (20 mL). $NEt_3$ (1.30 mL, 9.20 mmol, 3.30 eq.) was added and the mixture was stirred for 1 h at rt. The reaction mixture was washed with aqueous $NaHSO_4$ (5x15 mL, 0.5 M) and the organic phase were dried over $Na_2SO_4$. After filtration the solvent was evaporated, and the crude product dissolved in 1 mL DCM and 10 mL $H_2O$ were added. The biphasic mixture was sonicated for 30 min. This step was repeated once. The aqueous phase was decanted and the organic phase removed under reduced pressure, resulting in **S8** as a yellow oil (421 mg, 0.900 mmol, 32%).

**$^1$H-NMR** (300 MHz, $CDCl_3$): δ/ppm = 7.77-7.75 (d, *J* = 7.50 Hz, 2H), 7.61-7.55 (d, *J* = 7.40 Hz, 2H), 7.42-7.38 (t, *J* = 7.30 Hz, 2H), 7.33-7.28 (td, *J* = 7.40, 1.00 Hz, 2H), 4.47-4.41 (m, 2H), 4.26-4.22 (t, *J* = 6.60 Hz, 1H), 3.59-3.34 (m, 12H), 2.66 (br. s, 2H), 2.48-2-46 (m, 2H).

**$^{13}$C-NMR** (100 MHz, $CDCl_3$): δ/ppm = 177.54, 173.30, 158.29, 143.81, 141.48, 127.93, 127.24, 125.00, 120.16, 70.45, 70.31, 69.98, 69.63, 67.71, 47.25, 41.52, 39.57, 31.37, 30.38.

*The spectroscopic data was consistent with that reported in the literature.[4]*

**Synthesis of S9**

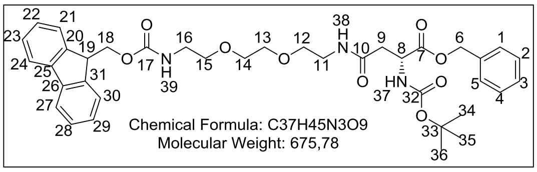


Based on a literature known procedure[5] Boc-Asp-OBzl (678 mg, 2,10 mmol, 1.00 eq.), HOBt (284 mg, 2.10 mmol, 1.00 eq.), EDC-HCl (651 mg, 4.20 mmol, 2.00 eq.) were dissolved in DCM (20 mL) at 0 °C and stirred for 30 min. Afterwards a solution of **S8** (1.00 g, 2.10 mmol, 1.00 eq.) in DCM (15 mL) and DiPEA (732 µL, 4.20 mmol, 2.00 eq.) were added to the reaction mixture and stirred for 3 h at rt. The reaction mixture was washed with aqueous citric acid (2x40 mL, 5%), Brine (2x40 mL) and the organic phase dried over $Na_2SO_4$. After filtration the solvent was removed *in vacuo*. Purification by column chromatography (EtOAc/DCM 4:1) yielded **S9** (1.00 g, 1.49 mmol, 71%) as a colorless, sticky solid.

**$R_f$** (EtOAc/DCM, 4:1) = 0.3

**$^1$H-NMR** (400 MHz, $CDCl_3$): δ/ppm = 7.77-7.76 (d, *J* = 7.5 Hz 2H, 24-H, 27-H), 7.60-7.58 (d, *J* = 7.4 Hz, 2H, 21-H, 30-H), 7.41-7.38 (t, *J* = 7.5 Hz, 2H, 22-H, 29-H), 7.34-7.29 (m, 7H, 1-H, 2-H, 3-H, 4-H, 5-H, 23-H, 28-H), 6.06 (s, 1H, 38-H), 5.81-5.79 (m, 1H, 37-H), 5.36 (s, 1H, 39-H), 5.20-5.12 (m, 2H, 6-H), 4.54 (s, 1H, 8-H). 4.43-4.41 (d, *J* = 6.9 Hz, 2H, 18-H), 4.23-4.20 (t, *J* = 6.7 Hz, 1H, 19-H), 3.61-3.46 (m, 8H, 12-H,13-H, 14-H, 15-H), 3.41-3.35 (m, 4H, 11-H, 16-H), 2.89-2.84 (m, 1H, 9-H), 2.69-2.63 (m, 1H, 9-H), 1.41 (s, 9H, 34-H, 35-H, 36-H).

**$^{13}$C-NMR**: (100 MHz, $CDCl_3$) δ/ppm = 171.54 (7-C), 169.93 (10-C), 156.63 (17-C), 155.82 (32-C), 144.02 (25-C, 26-C), 141.43 (20-C, 30-C), 128.59 (2-C, 4-C), 128.33 (3-C), 128.20 (1-C, 5-C), 127.82 (22-C, 29-C), 127.17 (23-C, 28-C), 125.12 (21-C, 30-C), 120.10 (24-C, 27-C), 79.99 (33-C), 70.38 (13-C), 70.17 (14-C), 69.73 (12-C, 15-C), 67.34 (6-C), 66.64 (18-C), 50.68 (8-C), 47.37 (19-C), 40.98 (11-C,), 39.31 (16-C), 37.86 (9-C), 28.41 (34-C, 35-C, 36-C).

**HRMS (ESI)**: m/z calcd. for $C_{37}H_{45}N_3O_9$+$Na^+$: 698.3048 [*M*+Na]$^+$; found: 698.3050.

**IR**: $\tilde{v}$=3325 (b), 2974 (w), 2935 (w), 2881 (w), 1685 (vs), 1647 (s), 1529 (vs), 1450 (s) 1365 (s), 1252 (vs), 1161 (vs), 1105 (vs) 1055 (s), 739 (vs), 698 (s) $cm^{-1}$.

**Synthesis of S10**

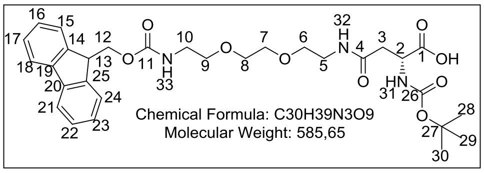


According to a literature known procedure[6], **S9** (270 mg, 0,400 mmol, 1.00 eq.) was dissolved in methanol (20 mL) and the reaction vessel was flushed with argon before Pd/C 10% (54.0 mg, 20 wt%) was added. Under vigorous stirring $Et_3SiH$ (638 µL, 4.00 mmol, 10.0 eq.) was added dropwise. After stirring for 10 min at rt, the solvent was evaporated under reduced pressure. The oily residue was dissolved in DCM (10 mL) and the organic phase washed with brine (2x20 mL). After drying over $Na_2SO_4$ the solvent was evaporated *in vacuo* and the residue was redissolved in DCM (1 mL). Hexane (10 mL) was added and the mixture was left to settle for 15 min. The supernatant was removed and the residue was evaporated to yield **S10** as a 11.5:1 mixture with $Et_3SiH$ (white solid, 140 mg, 0,239 mmol, 54%*).

**$^1$H-NMR** (400 MHz, $CDCl_3$): δ/ppm = 7.77-7.75 (d, $J$ = 7.50 Hz, 2H, 18-H, 21-H), 7.61-7.59 (d, $J$ = 7.80 Hz, 2H, 15-H, 24-H), 7.41-7.38 (t, $J$ = 7.40 Hz, 2H, 16-H, 23-H), 7.33-7.29 (t, $J$ = 7.50 Hz, 2H, 17-H, 22-H), 6.71 (s, 0.61H, 32-H), 5.88-5.84 (d, $J$ = 6.7 Hz, 1H, 31-H), 5.59 (s, 0.63H, 33-H), 4.46-4.41 (m, 3H, 2-H, 12-H), 4.23-4.21 (m, 1H, 13-H), 3.64-3.50 (m, 8H, 6-H, 7-H, 8-H, 9-H), 3.47-3.29 (m, 4H, 5-H, 10-H), 2.91-2.83 (m, 1H, 3-H), 2.68-2.59 (m, 1H, 3-H), 1.42 (s, 9H, 28-H, 29-H, 30-H).

**$^{13}$C-NMR**: (100 MHz, $CDCl_3$) δ/ppm = 172.61 (1-C), 171.73 (4-C), 156.75 (11-C), 155.64 (26-C), 144.01 (19-C, 20-C), 141.44 (14-C, 25-C), 127.85 (16-C, 23-C), 127.20 (17-C, 22-C), 125.16 (15-C, 24-C), 120.12 (18-C, 21-C), 80.32 (26-C), 70.36 (6-C, 9-C), 66.71 (12-C), 50.51 (2-C), 47.36 (13-C), 39.79 (5-C, 10-C), 38.12 (3-C), 28.43 (28-C, 29-C, 30-C).

**HRMS (ESI)**: m/z calcd. for $C_{30}H_{39}N_3O_9$+$Na^+$: 608.2579 [$M$+Na]$^+$; found: 608.2581.

**IR**: $\tilde{v}$=3323 (b), 2962 (w), 2929 (w), 2873 (w), 1699 (vs), 1660 (s), 1523 (s), 1450 (s), 1365 (m), 1248 (s), 1157 (vs), 1101 (s), 852 (w), 760 (s), 741 (vs) cm$^{-1}$.

**Melting point**: 67 °C

***Comment:*** *The yield was corrected for the $Et_3SiH$ impurity, which was removed after the next step, in which **S10** was used in excess.*

# 3. Physicochemical Experiments

## 3.1 Atomic Force Microscopy

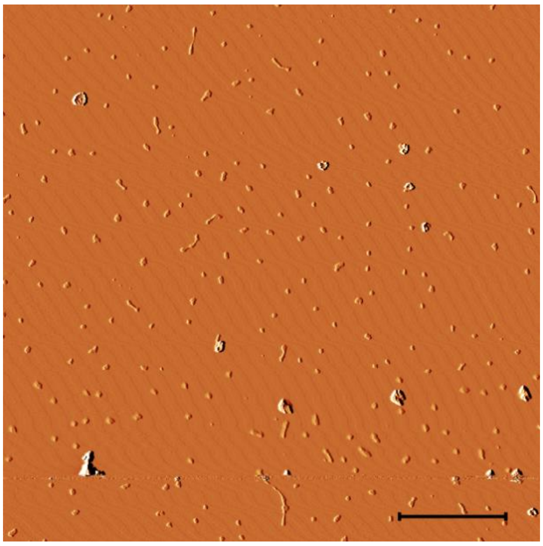

**Figure S1**. AFM image of plasmid DNA at 10 ug/mL after spincoating for 1 h. Scale bar represents 2 µm.

## 3.2 Dynamic Light Scattering

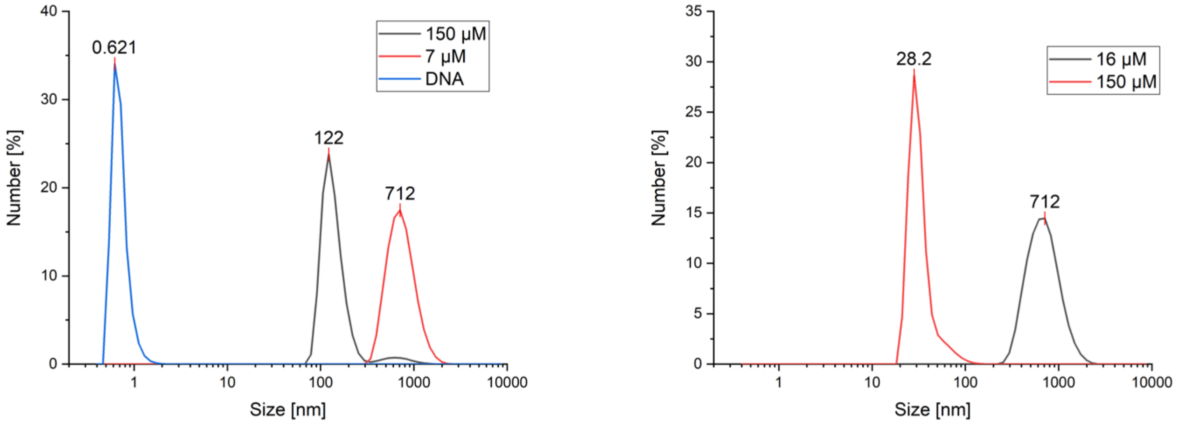


**Figure S2.** DLS titrations of **1** and **2** with DNA in $H_2O$ at given concentrations.

## 4. Biological experiments

### 4.1 Toxicity

Determination of the toxicity of the compounds was performed using the colorimetric CellTiter 96® AQueous One Solution Cell Proliferation Assay (Promega). Cells were seeded in a 96-well plate from (Greiner bio-one) 24 h prior to experiments at a confluence of 1 to 5 x104 (depending on the cell lines) in 100 µL of culture growth medium and grown at 37.0 °C, 5% CO2 atmosphere and 95% relative humidity. Cells were incubated for 5 h with the respective compound concentrations with the addition of plasmid DNA (1 µg/ml) to account for the transfection conditions. The proliferation assay was carried out according to the manufacturer's instructions. Absorbance at 490 nm was measured using a GloMax-multi plate reader (Promega). Raw data was processed, after subtracting the blank, by normalization to the DMSO control. Depicted is the value in percentage of the negative control which was termed cell viability.

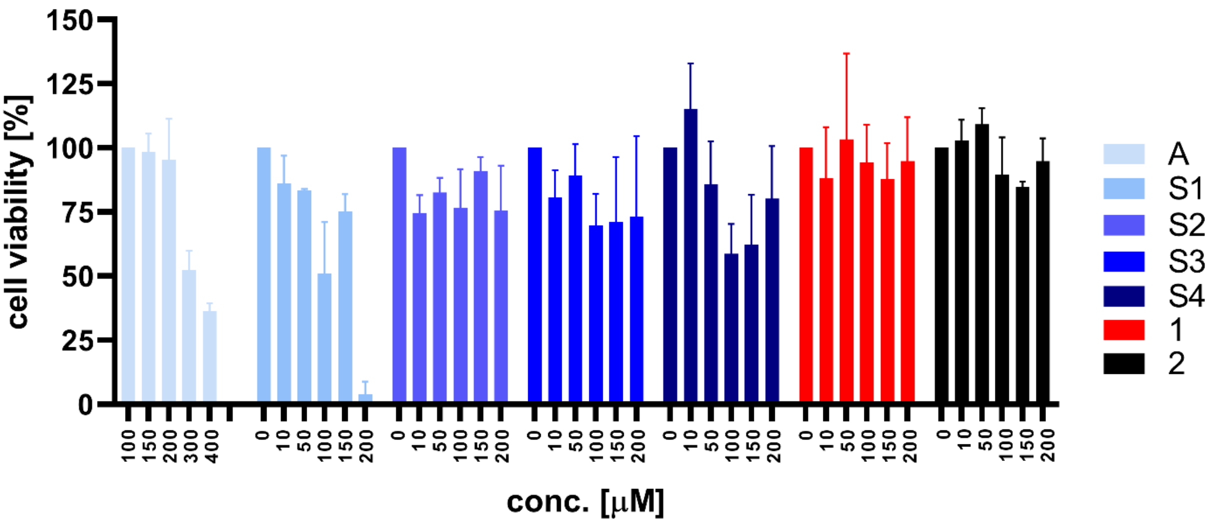


**Figure S3.** Toxicity of compounds **S1**, **S2**, **S3** and **S4** compared to **1** and **2** in HeLa cells. Compound A refers to the reference compound which is mentioned in Entry A Table 1.

### 4.2 Transfection and Microscopy

Cells were seeded in a 96-well plate from (*Greiner bio-one*) 24 h prior to experiments at a confluence of 1x$10^4$ in 100 µL of culture growth medium and grown at 37.0 °C, 5% $CO_2$ atmosphere and 95% relative humidity. The vector was dissolved in DMSO. 0.100 µg of DNA were diluted with DB-PBS and an appropriate amount of vector solution added resulting in a total volume of 30 µL. This solution was incubated for 20 min at rt. Afterwards 30.0 µL of culture growth medium was replaced by vector/DNA mixture. All precipitate which had formed during the incubation process was added to the well. After 5 h of incubation the cells were washed with warm DB-PBS buffer (2x100 µL per well) before incubating the cells for 24h at 37.0 °C, 5% $CO_2$ atmosphere and 95% relative humidity. After 24 h the cells were examined under the microscope.

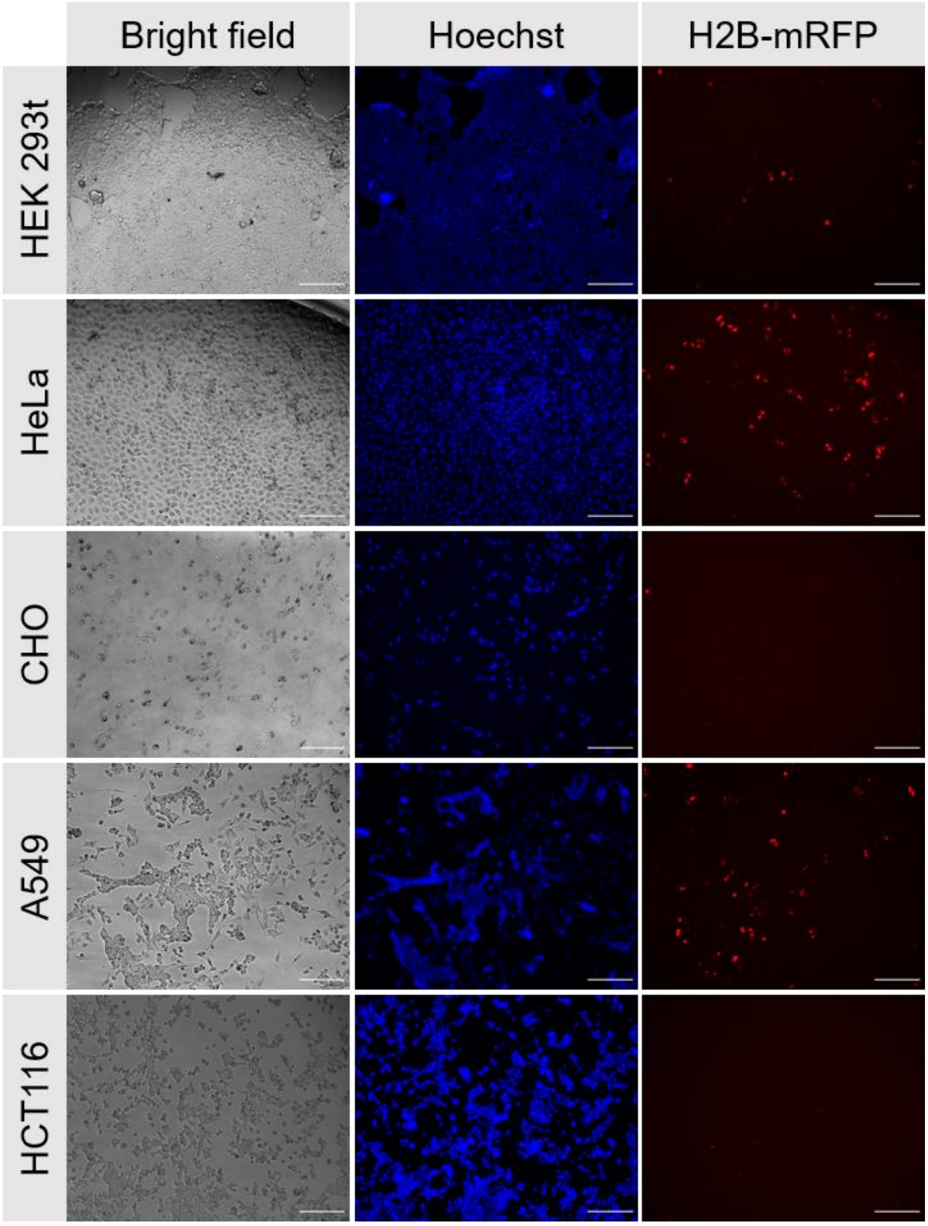


**Figure S4.** Representative microscopy images of HEK293t, HeLa, CHO, A549 and HCT116 cells 24 h after transfection with **1** (150 µM) with pH2B-mCherry. Scale bar represents 200 µm.

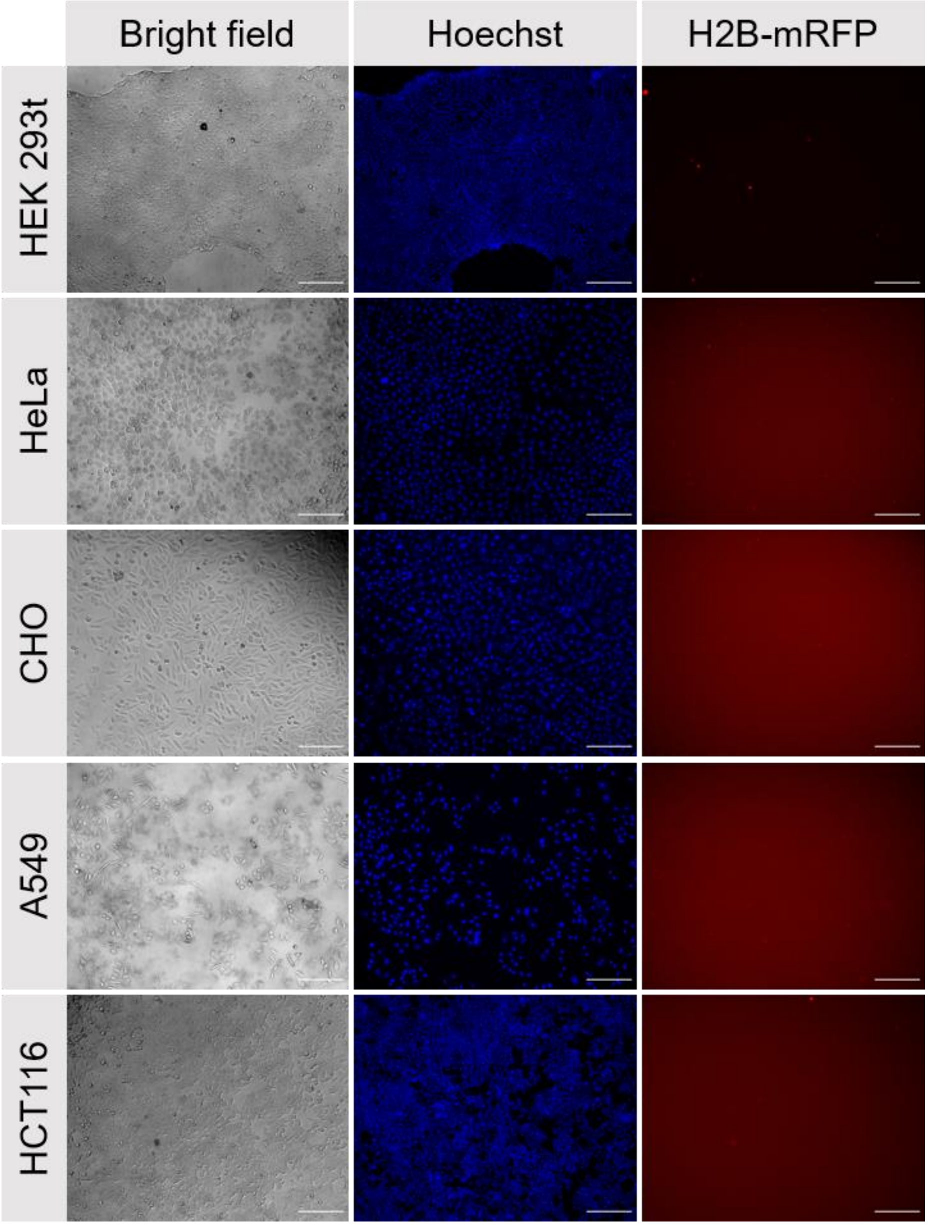


**Figure S5.** Representative microscopy images of HEK293t, HeLa, CHO, A549 and HCT116 cells 24 h after transfection with **2** (150 µM) with pH2B-mCherry. Scale bars represent 200 µm.

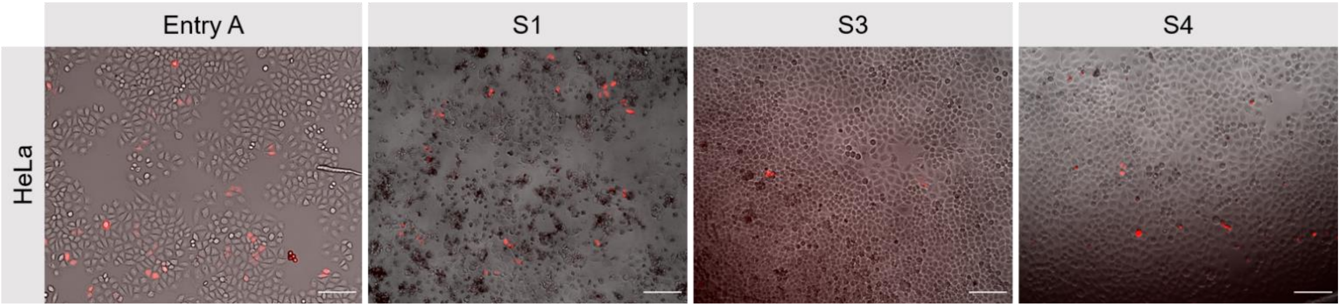


**Figure S6.** Representative microscopy images of HeLa cells 24 h after transfection with given compounds as quantified in Table 1 (Entry A: 150 µM, S1 400 µM, S3 200 µM, S4 150 µM) with pH2B-mCherry. Scale bars represent 200 µm.

### 4.3 Quantification of Transfection Results

For quantification of transfection efficiencies, cells were transfected with the pH2B-mCherry plasmid using the different compounds at the described concentrations and stained with DAPI. Images were acquired on a Zeiss Axio Observer 7 (Zeiss, Germany) using a Colibri 7 multi-color LED and a Zeiss AxioCam 506 mono CCD camera. The transfection efficiency was calculated using CellProfiler™.[7] First, nuclei were segmented based on the DAPI intensity (Ex = 385 nm; Em > 397 nm). Next, the fluorescence intensity in the red channel (Ex = 555 nm; Em > 590 nm) was measured within the segmented nuclei and a threshold was applied. The ratio between pH2B-mCherry positive nuclei and all nuclei (segmented by DAPI) was determined as transfection efficiency. Images were created using Fiji.[8]

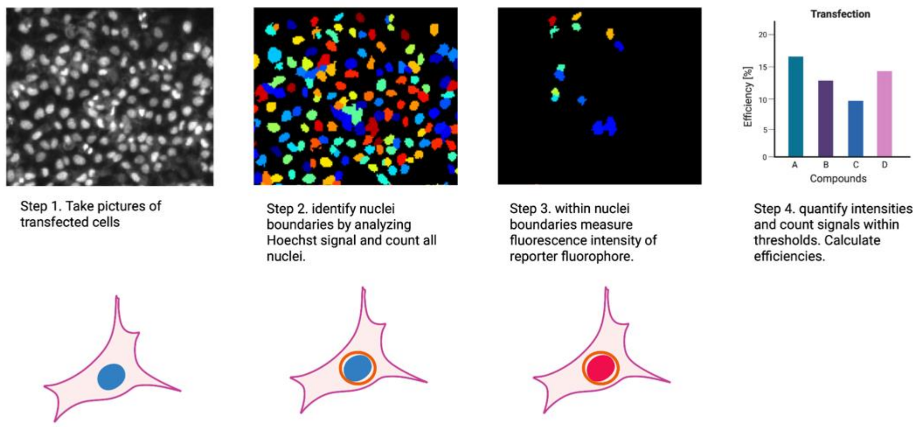


**Figure S7**. Flow chart of Cell Profiler® analysis of transfected cells. The pipeline recognizes Hoechst-stained nuclei and measures the intensity of the fluorescent reporter protein within the previously calculated nuclei boundaries.

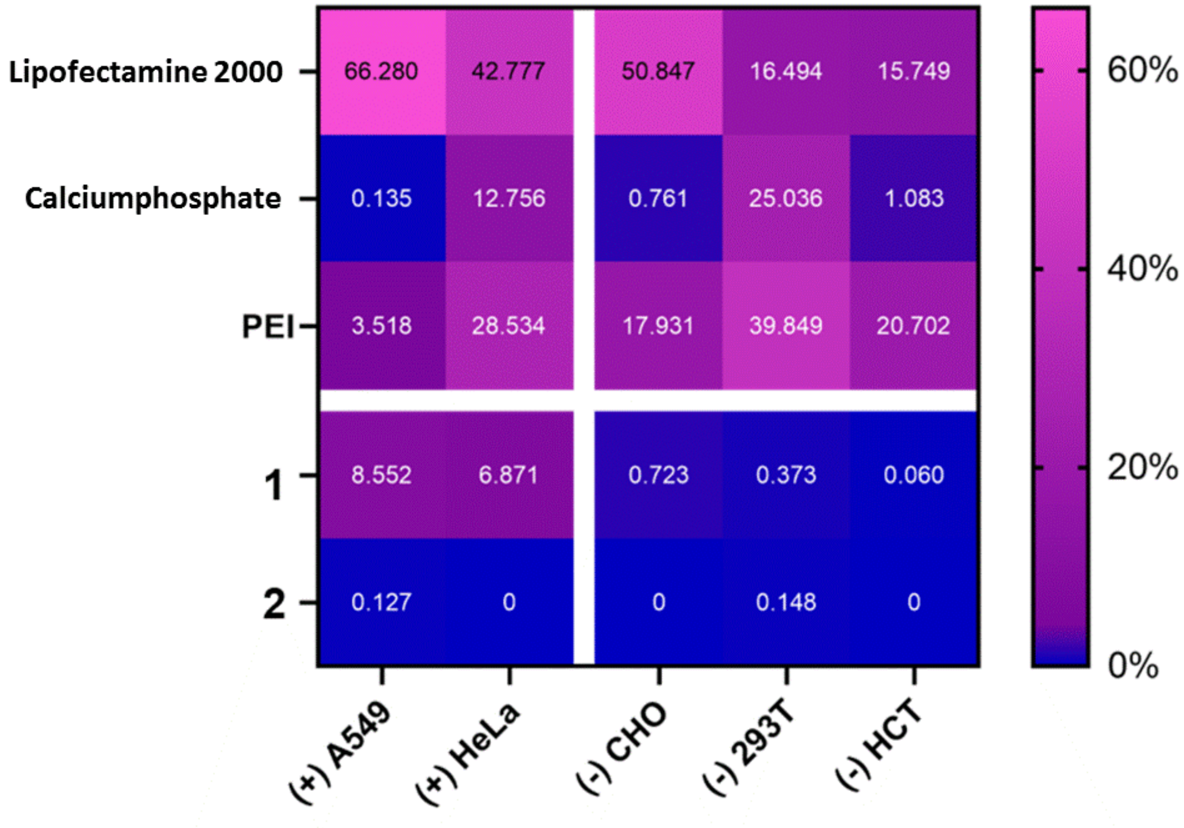


**Figure S8.** Heatmap representation of transfection efficiencies of vector **1** and **2** in comparison with Lipofectamine® 2000, calcium phosphate nanoparticles and linear PEI (25 kDa).

### 4.4 Biotin Competition Assay and Bafilomycin A1 Experiment

To gain insight into its mode of transfection, vector **1** was exposed to free biotin in increasing concentrations competing with **1** for biotin receptors (see Figure S7 B). HeLa cells were preincubated with Biotin-4-fluorescein in concentrations ranging from 0,075 to 1 mM. After 30 min of incubation (37.0 °C, 5% $CO_2$ atmosphere and 95% relative humidity) the transfection was carried out as described before, except that the concentration of **1** was increased to 300 µM and the amount of plasmid DNA was increased accordingly. This measure was taken to increase the level of competition between biotin and **1**. The cells were imaged 24 h after transfection. In addition, transfection under the influence of BafA1 was investigated (see Figure S7 B)). Bafilomycin A1 (BafA) interferes with the endocytic pathway, by causing alkalinization of the lumen and thus impair lysosomal function. HeLa cells were preincubated with BafA1 (100 nM). After 30 min of incubation (37.0 °C, 5% $CO_2$ atmosphere and 95% relative humidity) the transfection was carried out as described before.

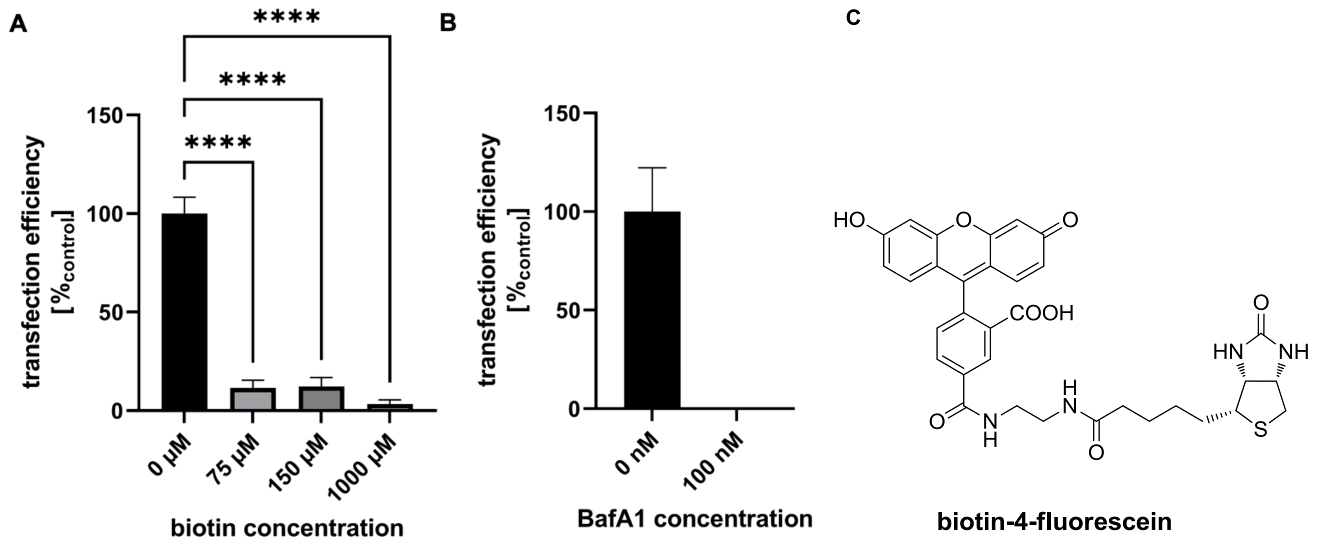


**Figure S9** A) Biotin competition assay: HeLa cells were preincubated with Biotin-4-fluorescein in concentrations as indicated. Transfection was performed using 300 µM of vector **1** and 0,2 µg plasmid DNA. Transfection efficiency was measured using Cell Profiler®. B) BafA1 effect assessment: HeLa cells were transfected using the previously described protocol using 150 µM **1** with and without preincubation of BafA1. C) Structure of biotin-4-fluorescein.

## 5. Compound Characterization

### HPLC and MS-spectra of S1

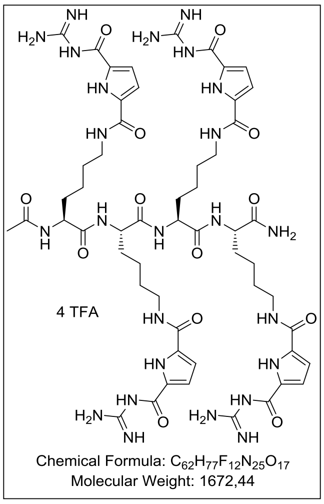


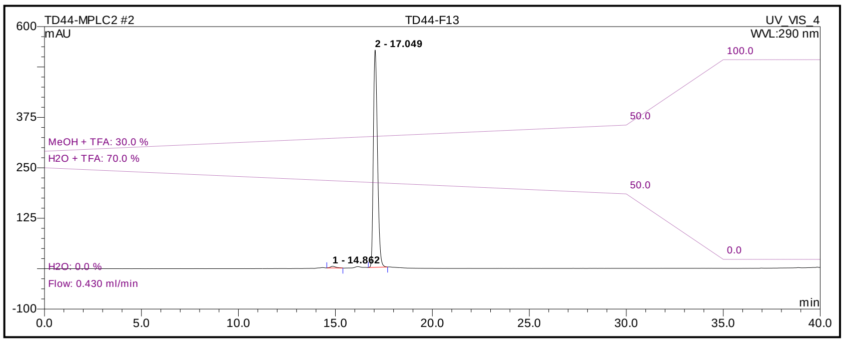


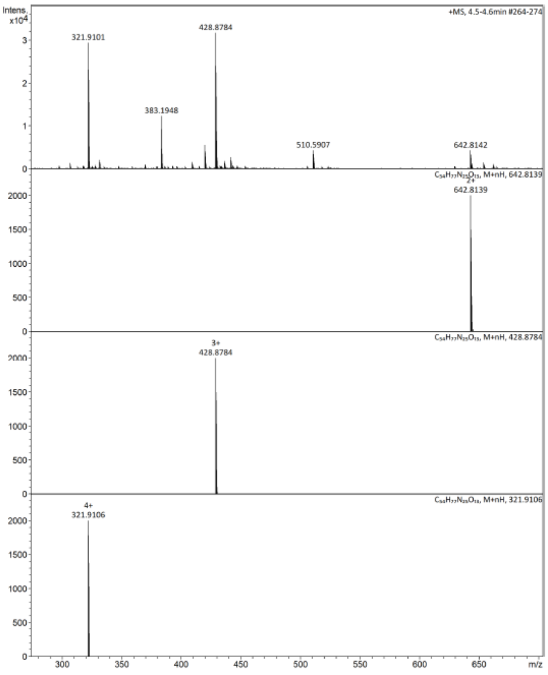

## HPLC and MS-spectra of S2

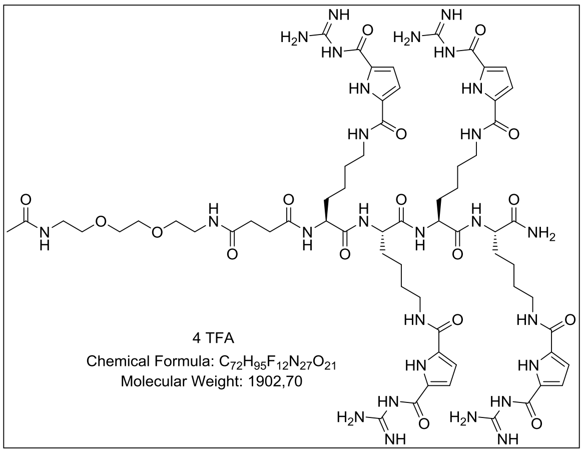


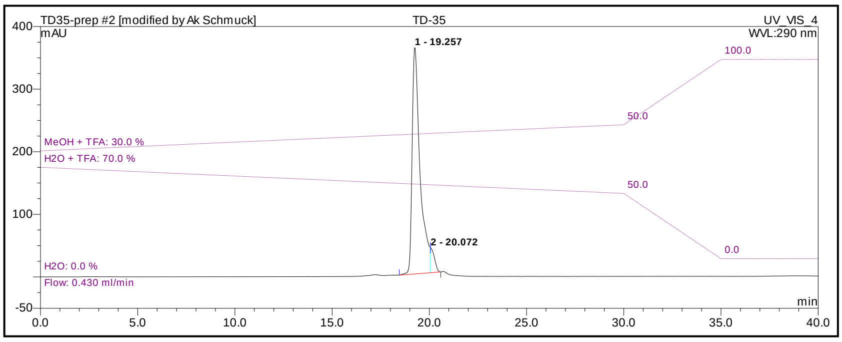


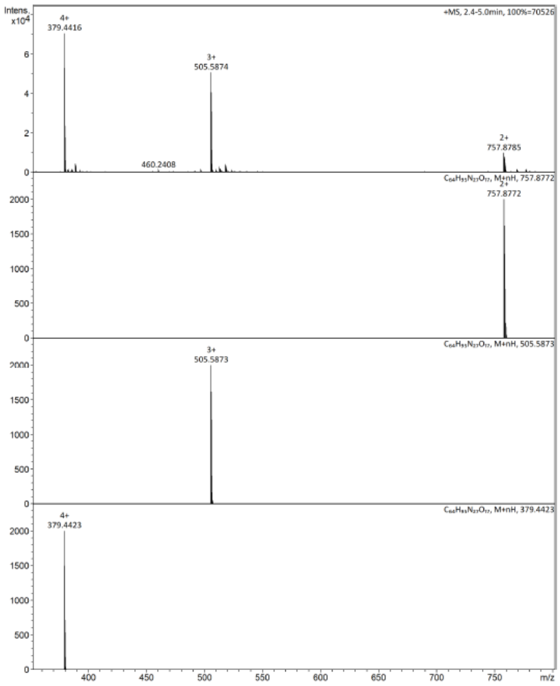

## HPLC and MS-spectra of S3

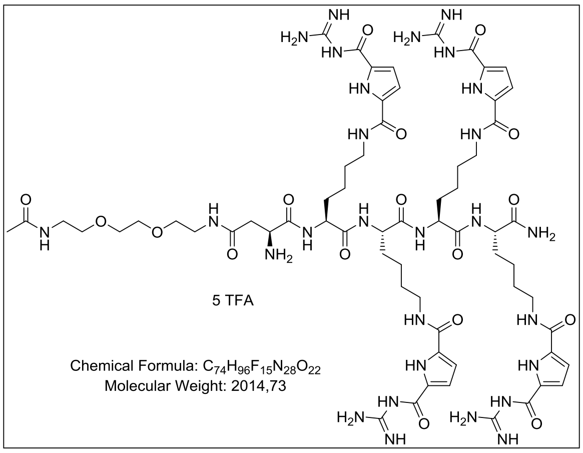


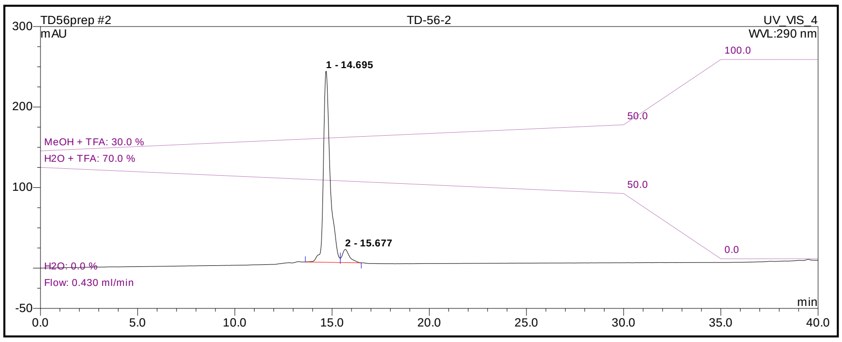


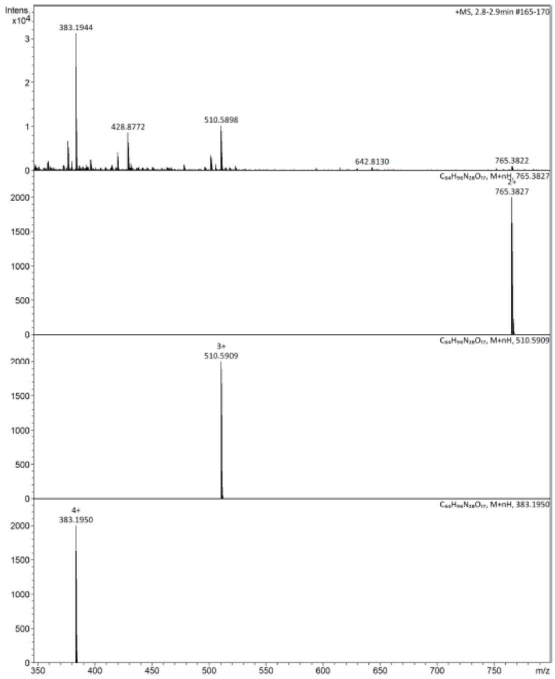

**HPLC and MS-spectra of S4**

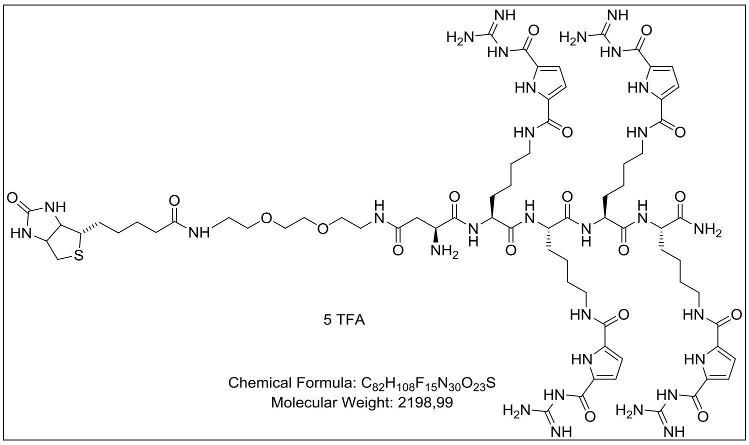


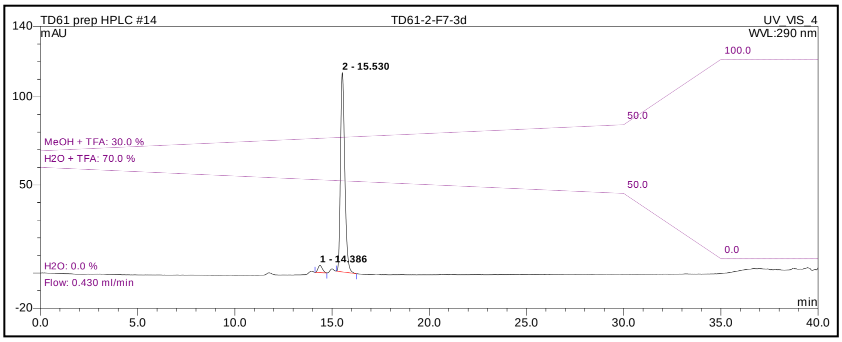


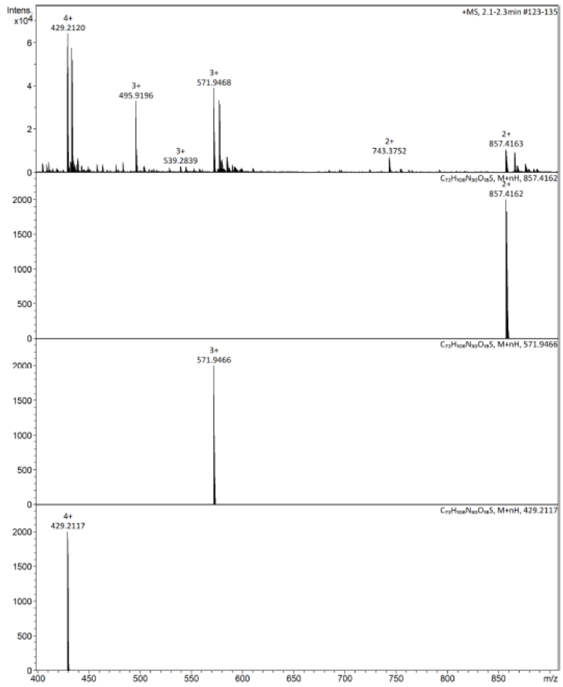

**NMR, HPLC and MS spectra of 1**

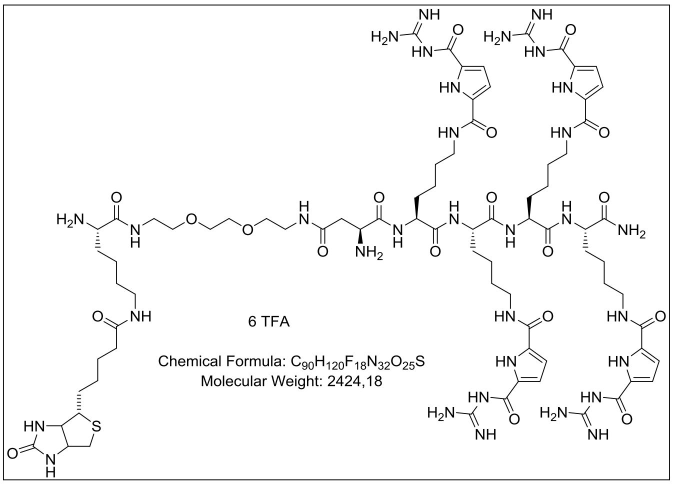


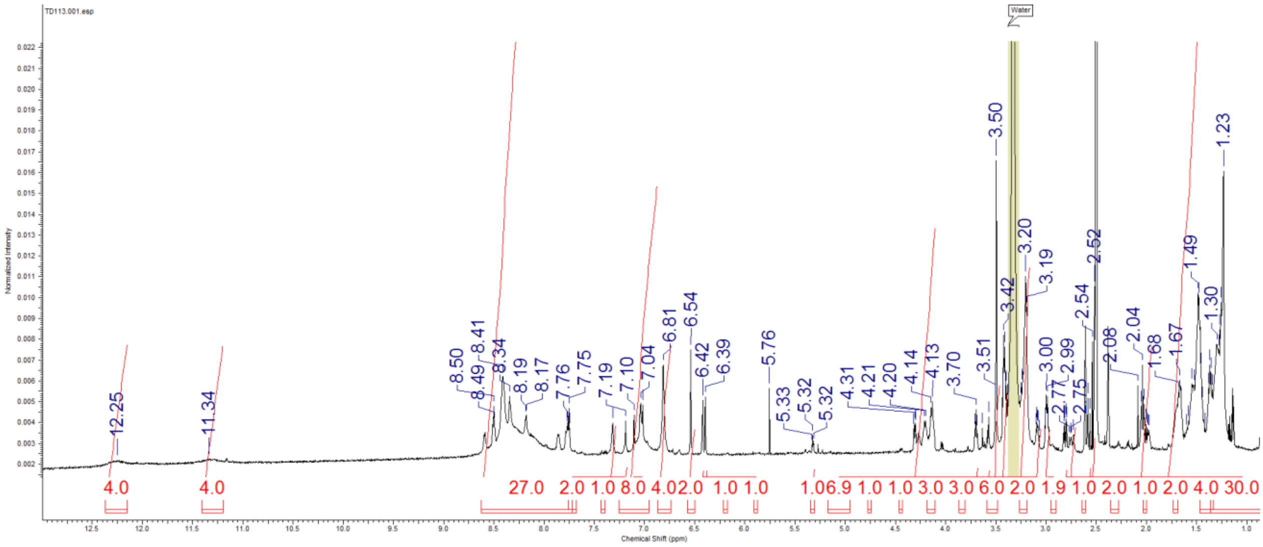


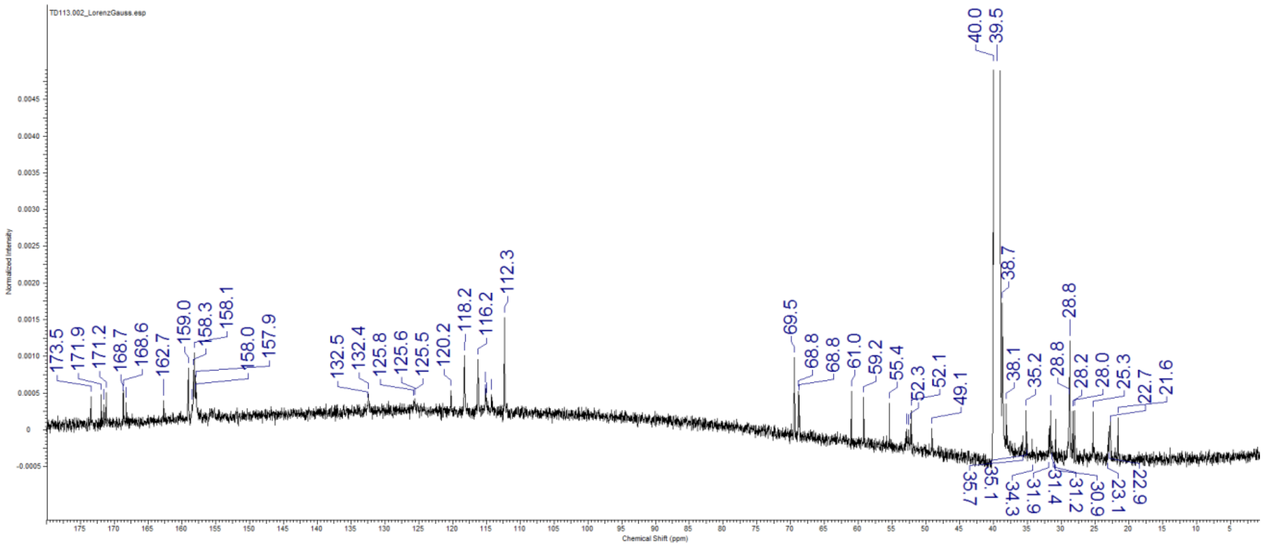


*$^{13}$C NMR Spectrum after application of a Lorenzian-Gaussian Window Function.*
*No Baseline correction was applied, as this led to the obscurance of key resonances.*

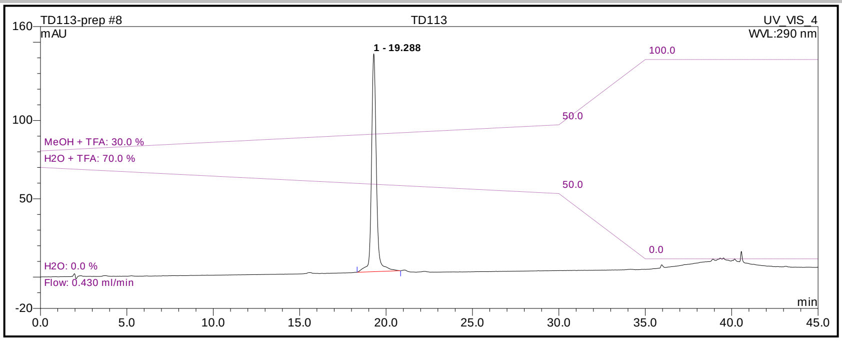
TD113-prep #8
TD113
UV_VIS_4
mAU
WVL:290 nm
1 - 19.288
100.0
50.0
MeOH + TFA: 30.0 %
H2O + TFA: 70.0 %
50.0
0.0
H2O: 0.0 %
Flow: 0.430 ml/min
min
160
100
50
-20
0.0
5.0
10.0
15.0
20.0
25.0
30.0
35.0
40.0
45.0

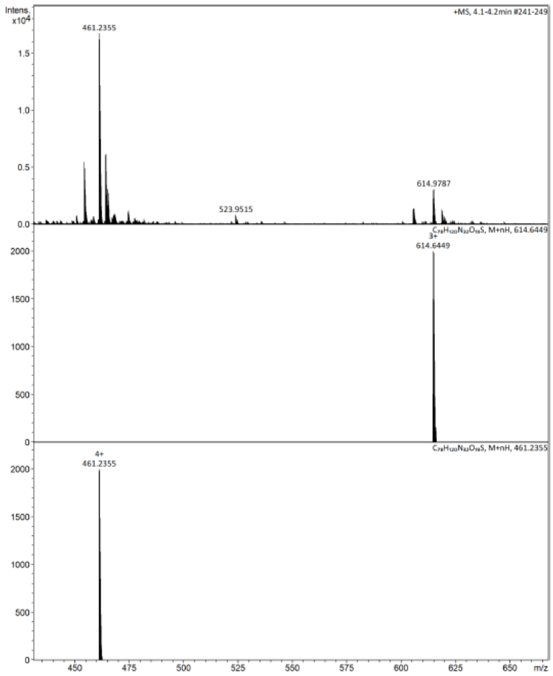
Intens.
x10^4
+MS, 4.1-4.2min #241-249
461.2355
523.9515
614.9787
M+nH, 614.6449
3+
614.6449
M+nH, 461.2355
4+
461.2355
1.5
1.0
0.5
0.0
2000
1500
1000
500
0
450
475
500
525
550
575
600
625
650
m/z

**NMR, HPLC and MS spectra of 2**

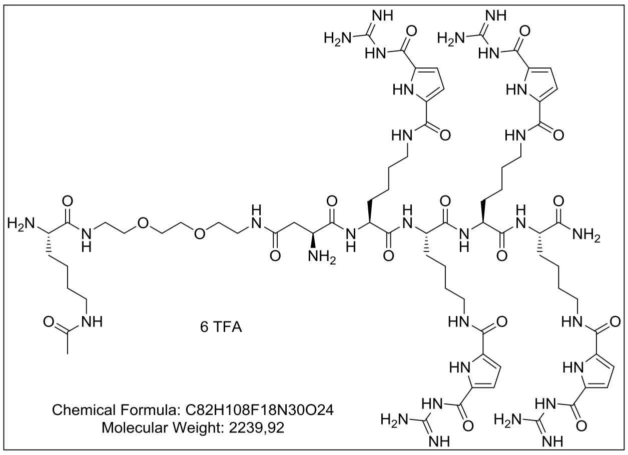


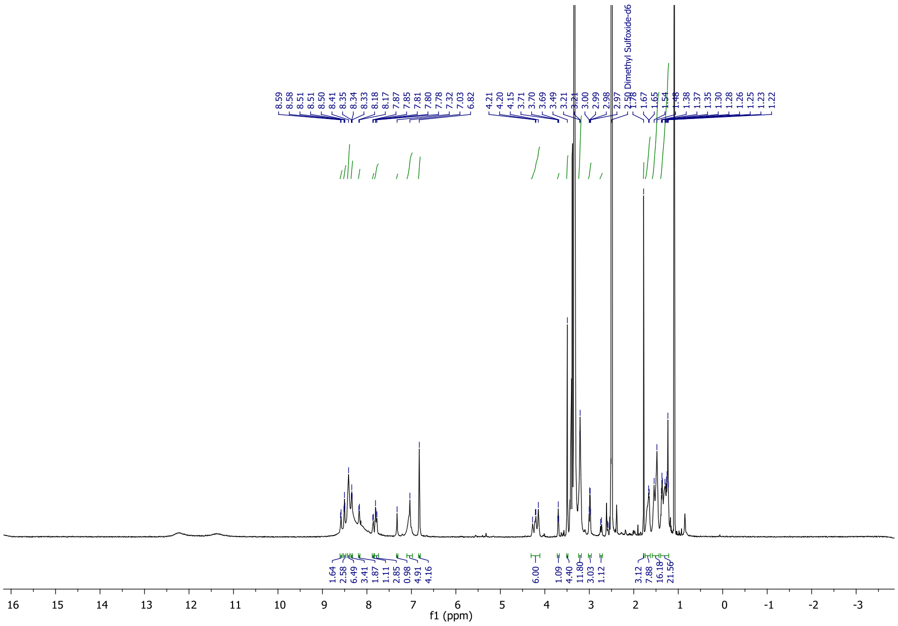


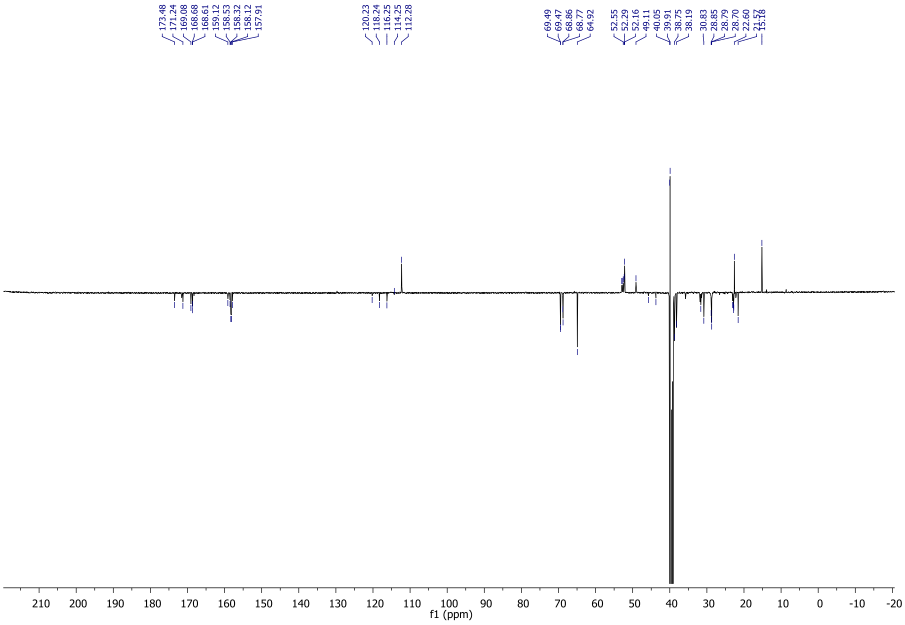

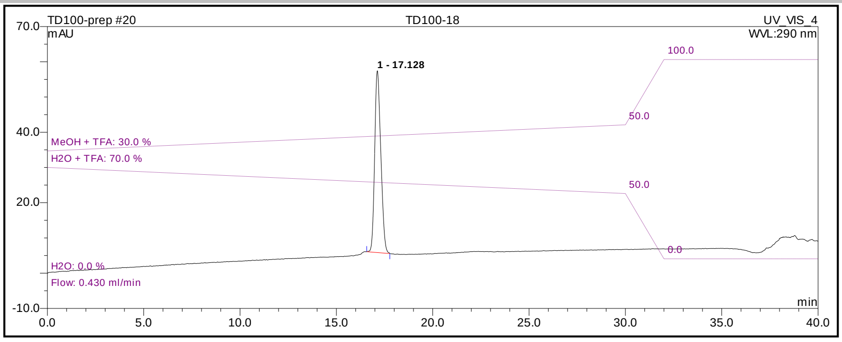
TD100-prep #20
TD100-18
UV_VIS_4
WVL:290 nm
mAU
1 - 17.128
MeOH + TFA: 30.0 %
H2O + TFA: 70.0 %
H2O: 0.0 %
Flow: 0.430 ml/min
100.0
50.0
50.0
0.0
70.0
40.0
20.0
-10.0
0.0
5.0
10.0
15.0
20.0
25.0
30.0
35.0
40.0
min

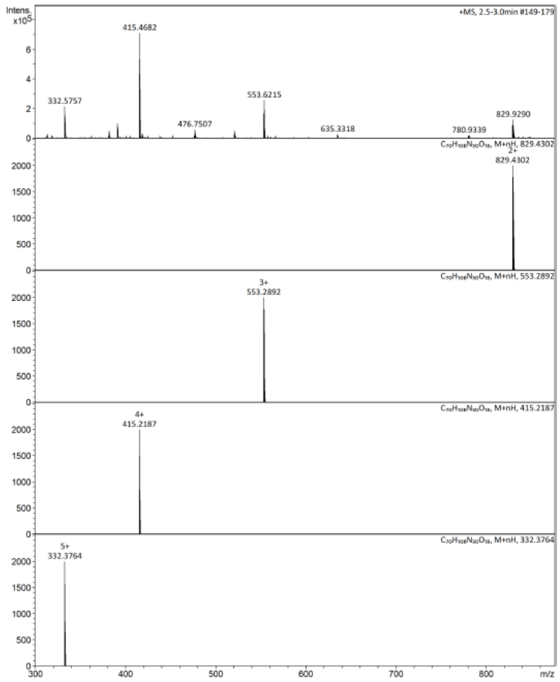
Intens.
x10^5
+MS, 2.5-3.0min #149-179
415.4682
332.5757
553.6215
476.7507
635.3318
780.9339
829.9290
C70H108N30O18, M+nH, 829.4302
2+
829.4302
C70H108N30O18, M+nH, 553.2892
3+
553.2892
C70H108N30O18, M+nH, 415.2187
4+
415.2187
C70H108N30O18, M+nH, 332.3764
5+
332.3764
300
400
500
600
700
800
m/z

**NMR spectra of S9**

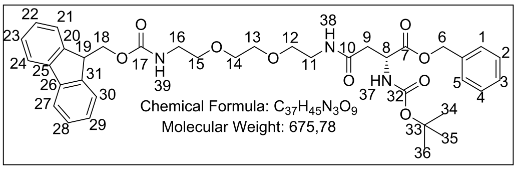


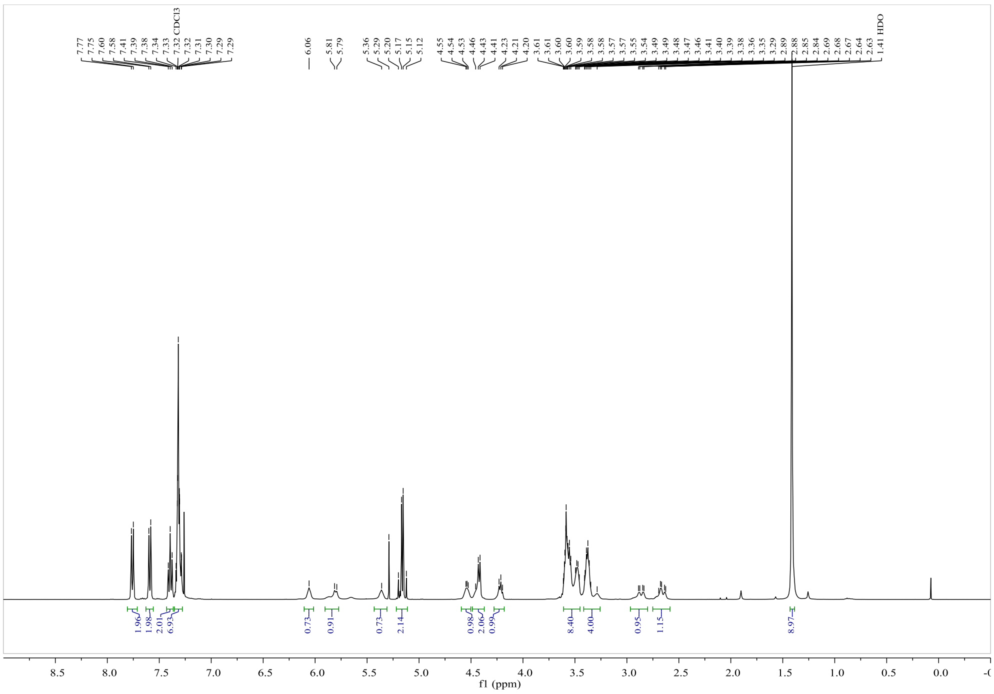


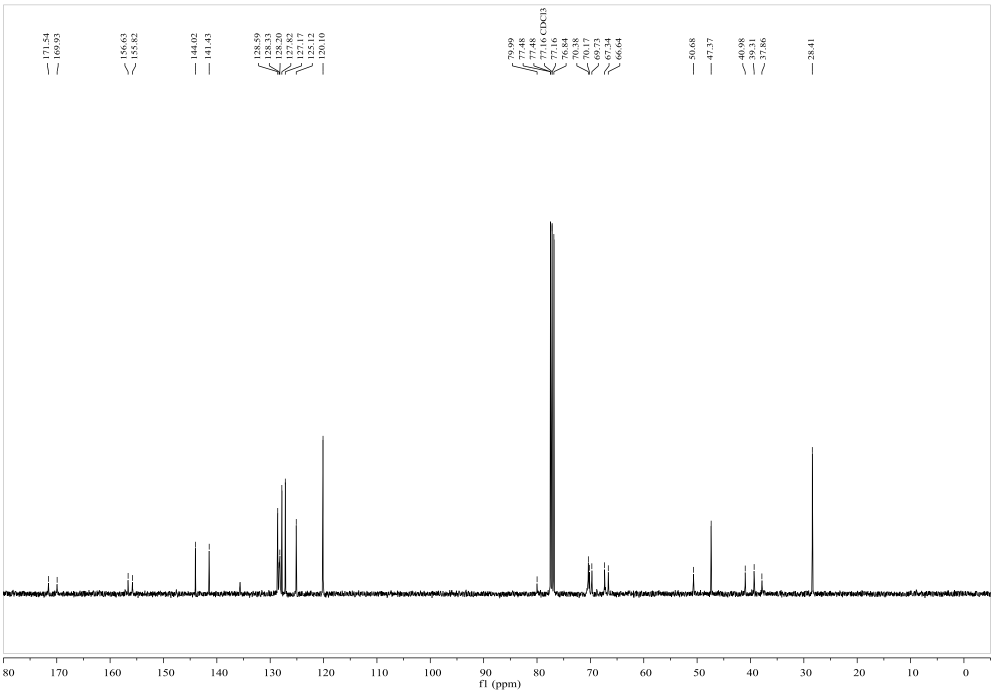

**NMR spectra of S10**

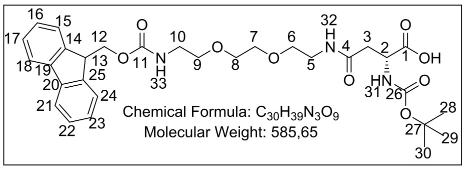


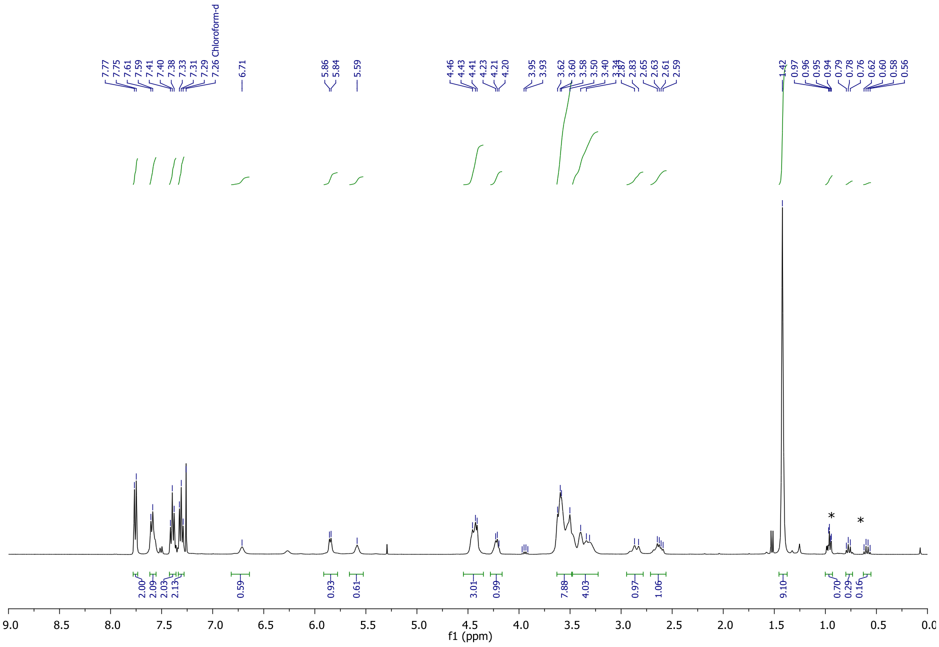

**$^*Et_3SiH$ (8%) Yield corrected for impurity**

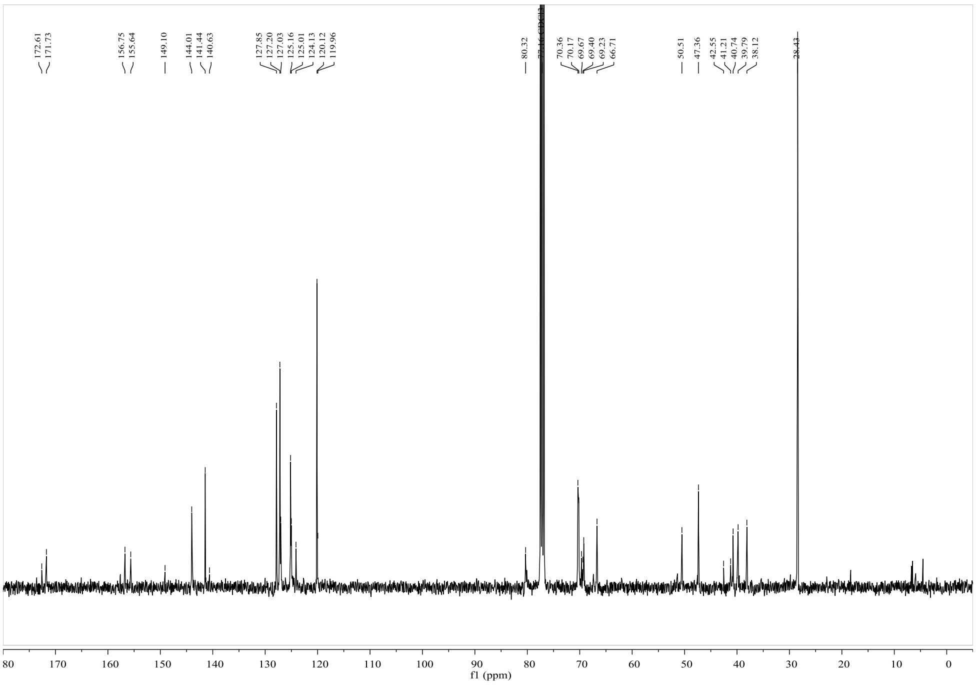

**Additional NMR spectra**

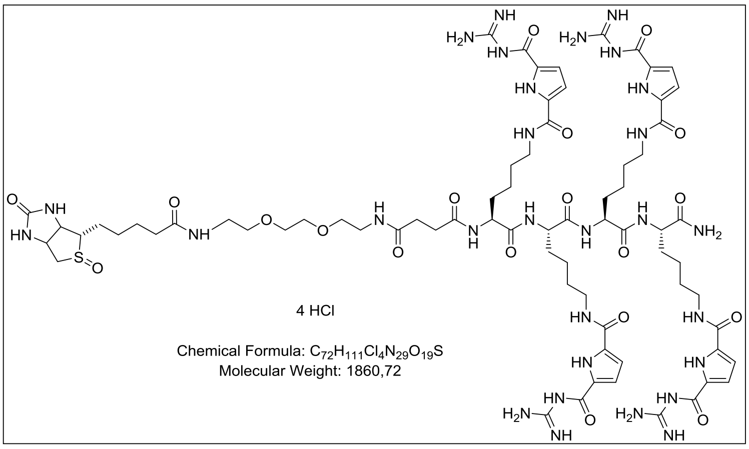


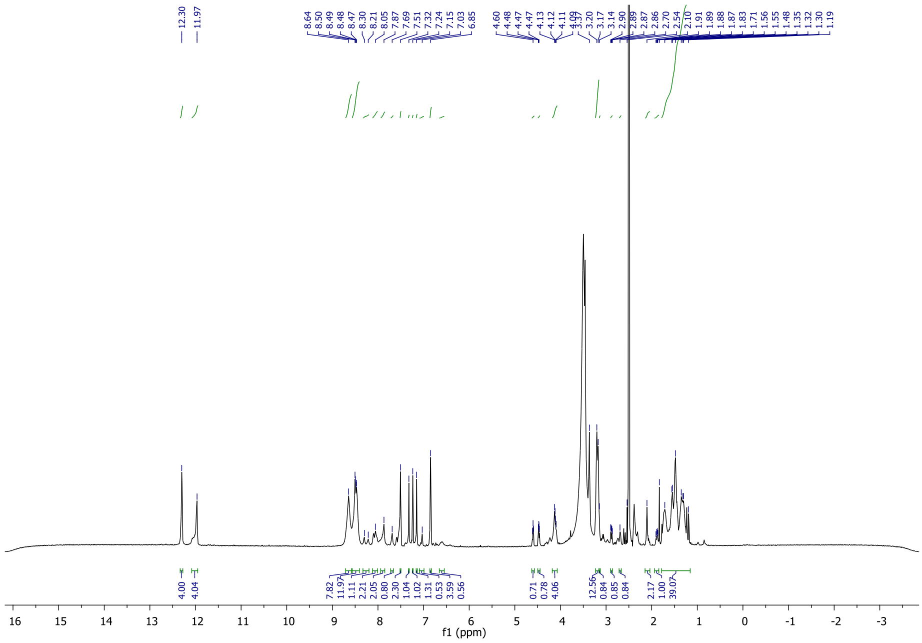


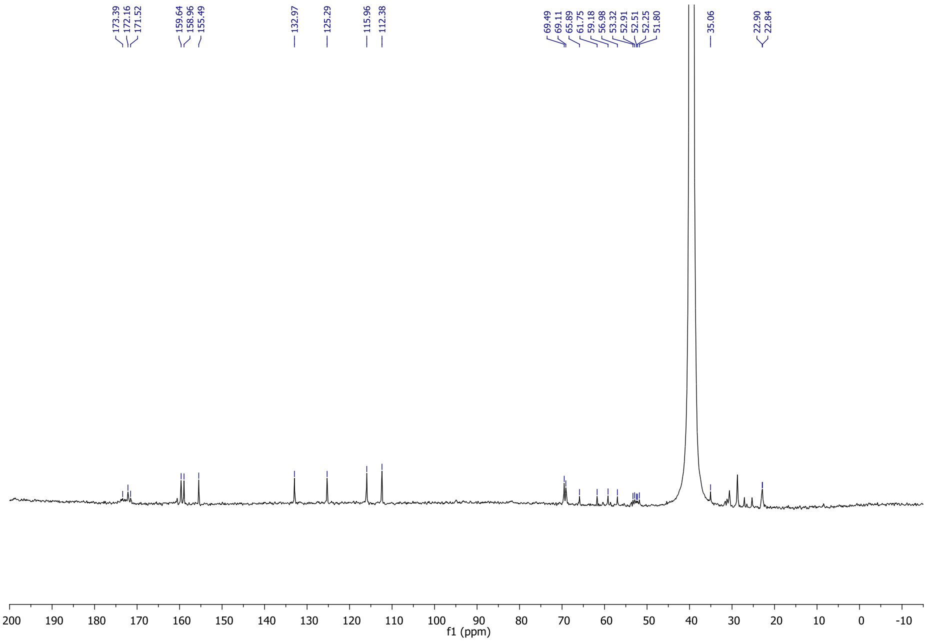

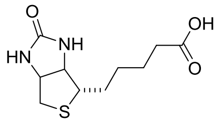


Chemical Formula: $C_{10}H_{16}N_2O_3S$
Molecular Weight: 244,31

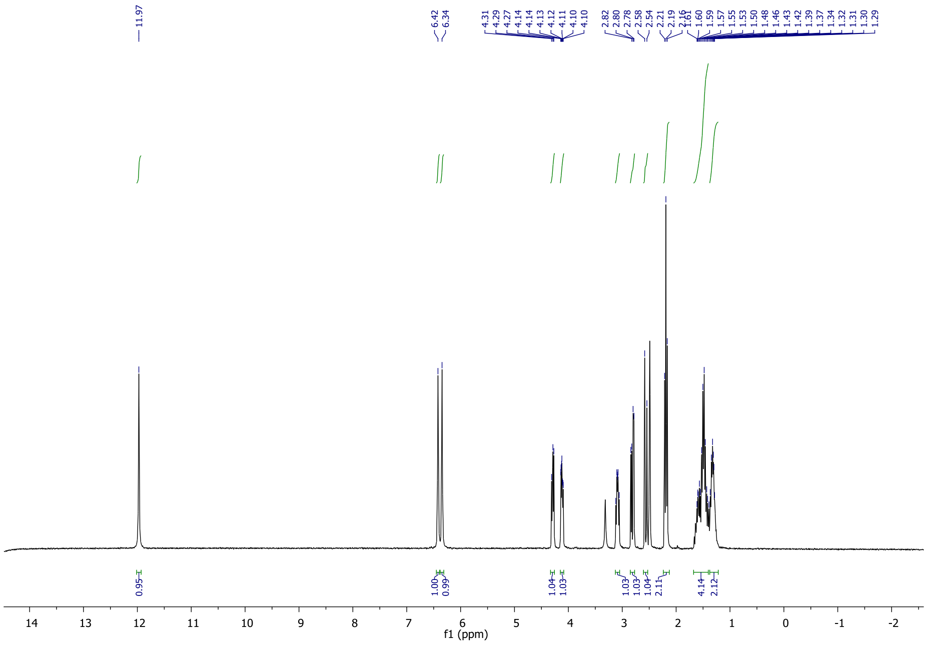


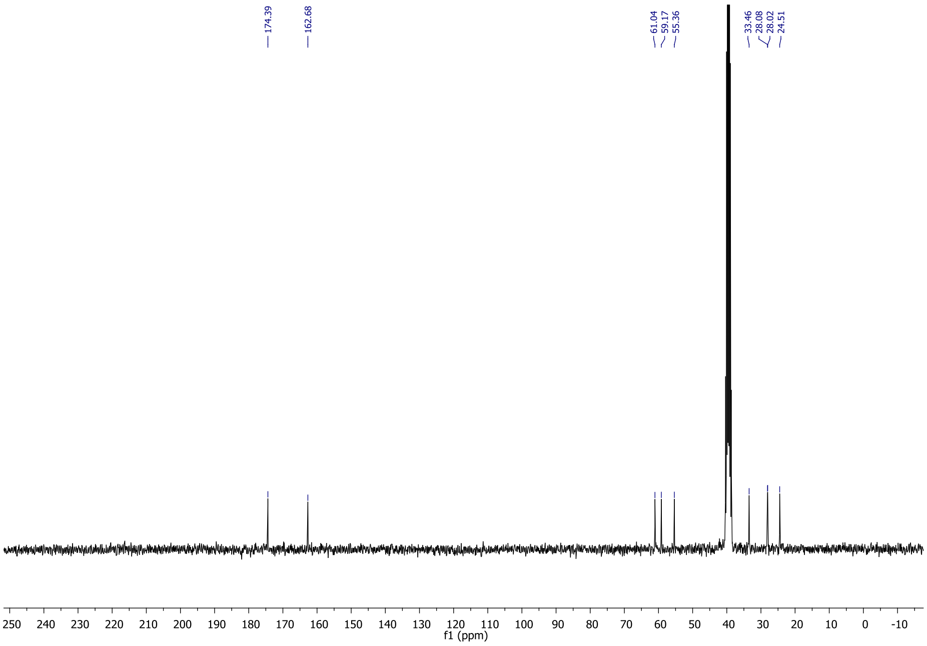

## References


[1] C. Schmuck, V. Bickert, M. Merschky, L. Geiger, D. Rupprecht, J. Dudaczek, P. Wich, T. Rehm, U. Machon, *Eur. J. Org. Chem.* **2008**, *2008*, 324.
[2] M. Ikura, K. Hikichi, *Org. Magn. Reson.* **1982**, *20*, 266.
[3] T. Otremba, B. J. Ravoo, *Tetrahedron* **2017**, *73*, 4972.
[4] D. Ponader, F. Wojcik, F. Beceren-Braun, J. Dernedde, L. Hartmann, *Biomacromolecules* **2012**, *13*, 1845.
[5] R. Zhuang, L. Gao, X. Lv, J. Xi, L. Sheng, Y. Zhao, R. He, X. Hu, Y. Shao, X. Pan et al., *Eur. J. Med. Chem.* **2017**, *126*, 1056.
[6] P. K. Mandal, J. S. McMurray, *J. Org. Chem.* **2007**, *72*, 6599.
[7] A. E. Carpenter, T. R. Jones, M. R. Lamprecht, C. Clarke, I. H. Kang, O. Friman, D. A. Guertin, J. H. Chang, R. A. Lindquist, J. Moffat et al., *Genome Biol.* **2006**, *7*, R100.
[8] J. Schindelin, I. Arganda-Carreras, E. Frise, V. Kaynig, M. Longair, T. Pietzsch, S. Preibisch, C. Rueden, S. Saalfeld, B. Schmid et al., *Nat. Methods* **2012**, *9*, 676.


## Author Contributions


**T. Dirksmeyer:** Compound synthesis and characterization, AFM, DLS and transfection experiments, data analysis, manuscript preparation (writing of the original draft) and funding acquisition.

**P. Stahl:** Support and design of biological assays, optimization of the transfection protocol, cultivation and seeding of cell lines, toxicity experiments, data analysis and manuscript preparation.

**C. Vallet:** Support and design of biological assays, proof reading.

**S. Knauer:** Supervisor (Biology) and manuscript preparation.

**M. Giese:** Project head after 2019 and proof reading.

**C. Schmuck:** Project head until 2019 and funding acquisition.

**C. Hirschhäuser:** Supervisor (Chemistry) and manuscript preparation.
